# The interplay between *Helicobacter pylori* and gastrointestinal microbiota

**DOI:** 10.1080/19490976.2021.1909459

**Published:** 2021-05-03

**Authors:** Chieh-Chang Chen, Jyh-Ming Liou, Yi-Chia Lee, Tzu-Chan Hong, Emad M El-Omar, Ming-Shiang Wu

**Affiliations:** a Division of Gastroenterology and Hepatology, Department of Internal Medicine, National Taiwan University Hospital, Taipei, Taiwan; b Department of Internal Medicine, National Taiwan University College of Medicine, Taipei, Taiwan; c Graduate Institute of Clinical Medicine, National Taiwan University College of Medicine, Taipei, Taiwan; d Department of Medicine, National Taiwan University Cancer Center, National Taiwan University College of Medicine, Taipei, Taiwan; e Department of Medical Research, National Taiwan University Hospital, Taipei, Taiwan; f Department of Internal Medicine, National Taiwan University Hospital, Taipei, Taiwan; g Microbiome Research Centre, St George & Sutherland Clinical School, University of New South Wales, Sydney, NSW, Australia

**Keywords:** *Helicobacter pylori*, esophageal microbiota, gastric microbiota, gastric cancer, colonic microbiota, eradication therapy

## Abstract

The complex population of microbes in the human gastrointestinal (GI) tract interacts with itself and with the host, exerting a deep influence on health and disease development. The development of modern sequencing technology has enabled us to gain insight into GI microbes. *Helicobacter pylori* colonization significantly affects the gastric microenvironment, which in turn affects gastric microbiota and may be correlated with colonic microbiota changes. Crosstalk between *H. pylori* and GI commensal flora may play a role in *H. pylori*–related carcinogenicity and extragastric manifestations. We review current knowledge on how *H. pylori* shapes GI microbiota with a specific focus on its impact on the stomach and colon. We also review current evidence on colonic microbiota changes attributed to eradication therapy based on the clinical studies performed to date.

## Introduction

Trillions of microorganisms reside in the human gastrointestinal (GI) tract and form a symbiotic relationship with the host, playing an important role in health and disease. The GI microbiome and the host generate a complex network of interactions that transcends the boundaries of the GI tract, forging intimate connections with all aspects of human physiology, including metabolic, immune, and neuroendocrine systems. The crosstalk is mediated by microbial-derived biochemical signals that are absorbed into the blood and circulated throughout the human body; by signals relayed by the enteric nervous system that transmit microbiota-derived cues to the central nervous system; and by immune cells that perceive local microbial signals in the GI tract and are trafficked throughout the body.^1–3^

As a GI tract microbe, *Helicobacter pylori* is one of the most-studied bacteria. It is highly adapted to the human gastric mucosa and thrives in the stomach niche, having co-evolved with humans over tens of thousands of years.^4^ Chronic infection can lead to either hypo- or hyperchlorhydria, depending on the anatomic distribution and severity of the resulting inflammation.^5^ Although the majority of *H. pylori*–infected persons remain asymptomatic, chronic infection has been linked to peptic ulcer disease, gastric cancer, gastric mucosa-associated lymphoid tissue lymphoma, and a multitude of extragastric diseases. Current studies suggest that eradication of *H. pylori* can effectively reduce gastric cancer incidence and treatment should be considered for all *H. pylori*–infected persons to reduce the risk of peptic ulcers and gastric cancers.^6–8^ However, there are still debates regarding the beneficial effects of *H. pylori* colonization, including regression in childhood asthma and other atopic disorders.^9,10^ It has been concluded that *H. pylori* is a common flora, or at least a harmless bacterium. Additionally, the mass eradication of *H. pylori* with antibiotic treatment as a preventive measure for gastric cancer and peptic ulcers raises several concerns, including the emergence of antibiotic resistance and perturbations in gut microbiota following *H. pylori* eradication.^11,12^ Being part of the GI ecosystem, *H. pylori* infection and its impact on gastric acid secretion may alter the GI microbiome and host health status. Here, we review current understandings of the impact of *H. pylori* infection on the GI microbiome and how it inﬂuences human health.

## *Helicobacter pylori* and the esophageal microbiome

### The esophageal microbiome in the normal esophagus

Although the esophagus serves as the beginning of the digestive tract, the esophageal microbiome has long been overlooked and little is known about it relative to our understanding of the composition and function of the gut microbiome. Early culture-based studies using esophageal washing demonstrated a high proportion of *Streptococcus viridans* and a pattern resembling that of the oral microbiome.^13,14^ The first culture-independent investigation of the distal esophageal microbiome identified a far more complex microbial community, comprising six major phyla (Firmicutes, Bacteroides, Actinobacteria, Proteobacteria, Fusobacteria, and TM7), with *Streptococcus* as the most prevalent genus.^15^ *Streptococcus, Hemophilus, Neisseria, Prevotella*, and *Veillonella* are considered to be the core microbes in the normal esophagus.^16^ However, bacterial composition may differ depending on various factors, such as age, use of proton pump inhibitors, and disease.^16–18^

### The esophageal microbiome in reflux esophagitis, Barrett’s esophagus, and esophageal adenocarcinoma

Chronic gastric acid exposure or duodenal bile in the distal esophagus is considered to be the primary factor in the pathogenesis of reflux esophagitis. It was widely accepted that reflux may cause chronic esophageal injury and promote carcinogenesis in Barrett’s esophagus. A culture-independent study by Yang et al. classified the esophageal microbiota into two distinct types.^19^ The healthy esophagus harbored Gram-positive taxa from the Firmicutes phylum, of which *Streptococcus* was the dominant genus (Type I microbiome), while an inflamed esophagus (reflux esophagitis or Barrett’s esophagus) was dominated by Gram-negative taxa from the Bacteroidetes, Proteobacteria, and Fusobacteria phyla (Type II microbiome). These findings are consistent with other studies,^18,20,21^ reliably demonstrating a change in esophageal microbiota in cases of reflux disease that most likely reflects physiological changes due to excess gastric acid. Studies investigating the microbiota in cases of esophageal adenocarcinoma (EAC) are rare. The studies by Elliott et al. and Snider et al. identified reduced microbial diversity in EAC samples compared with controls.^22,23^ Some EAC samples were dominated by a single bacterial species belonging to the order Lactobacillales in the study by Elliott et al., while Snider et al. found more Enterobacteriaceae and *Akkermansia muciniphila* in patients with high-grade dysplasia or EAC. Both studies had relatively small sample sizes and further research is required before an EAC microbiome signature can be defined.

### Helicobacter pylori, the esophageal microbiome, and esophageal diseases

The incidences of gastroesophageal reflux disease, Barrett’s esophagus, and EAC have been rising over the past several decades in developed countries and are inversely associated with *H. pylori* infection prevalence.^24–27^ Previous research describes the existence of a core esophageal microbiota and has shown that its composition in healthy controls differs at the phylum and genus levels from patients with reflux esophagitis or Barrett’s esophagus. The altered bacterial microenvironment may contribute substantially to esophageal mucosa injury and further carcinogenesis. One of the hypotheses explaining the protection by *H. pylori* against Barrett’s esophagus and EAC may relate to the fact that at the population-level it reduces acid secretion. *H. pylori* also influences colonization by other important organisms. Amir et al. and Deshpande et al. determined that the administration of proton pump inhibitors influences microbial composition in the esophagus, and this effect is thought to be related to acid levels.^18,28^ The *H. pylori*–positive stomach produces less acid and the microbial community in the distal esophagus is probably altered when reflux occurs. It would be interesting to determine whether *H. pylori* interacts with the esophageal microbiota to confer protection against Barrett’s esophagus or EAC. However, this is a current gap in esophageal microbiome research, and no studies have assessed whether hosts’ *H. pylori* status contributes to different esophageal microbial communities. It is imperative to study the impact of *H. pylori* on host physiology and the ensuing effect on the esophageal microbiome, although this may become increasingly difficult due to a declining prevalence of *H. pylori*.

## *Helicobacter pylori* and the gastric microbiome

### The normal gastric microbiome

Although Gillespie isolated 24 different organisms from the stomach through a stomach tube in 1893, the stomach was still considered sterile due to its acidic environment. Microbes cultured from gastric fluid were generally considered to be transient or passing luminal microbes until the discovery of *H. pylori* in 1982.^29^ For the next few decades, *H. pylori* was considered to be the only organism capable of surviving in the hostile gastric environment because culturing was the mainstay of microbial research.^30,31^ However, the majority of bacteria are difﬁcult to culture or are uncultivable.

Culture-independent methods, particularly next-generation sequencing (NGS) technology, have broadened the horizons in human microbial research.^32^ Studies employing NGS reveal that human gastric microbes are more diverse than initially anticipated.^33–35^ Published studies show significant heterogeneity of gastric microbiota, which may be attributed to inter-individual variability, ethnicity, different sample types, different gastric pathologies, and the use of different technical approaches. In a review article, Rajilic‐Stojanovic et al. compared the studies that investigated the gastric microbiota using NGS. Based on an arbitrary cut‐off value requiring genera to be present in more than 20% of the included studies, the typical gastric microbiota consists of 57 bacterial genera distributed among eight phyla, including Actinobacteria, Bacteroidetes, Firmicutes, Fusobacteria, Proteobacteria, Spirochetes, Tenericutes, and TM7.^36^ The six most common genera reported were *Prevotella, Streptococcus, Neisseria, Hemophilus, Fusobacterium*, and *Veillonella. Helicobacter* was detected in 23 of 36 studies. The bacterial community of the normal stomach has not been extensively characterized; only four studies have reported on the microbiota present in healthy adults, and these provide us with a snapshot of healthy gastric microbiota.^37–40^ All studies reported the presence of *Prevotella, Streptococcus, Megasphaerae, Capnocytophaga, Oribacterium*, and *Propionibacterium*. It is noteworthy that around half of the 266 reported genera were only found in one study, indicating that these groups are most likely of low biological relevance or due to artifacts from the sequencing technique or bioinformatic processing.^41^

### Effect of Helicobacter pylori on the gastric microbiome

*H. pylori* employs several enzymatic machineries that permit its survival in the harsh acidic conditions of the stomach.^42^ When *H. pylori* is present, it is the most abundant organism of the gastric microbiota, representing 40%–90% of the gastric microbiota.^34,43–47^ The alpha diversity of bacteria in the stomach is negatively associated with the presence of *H. pylori*.^34,43,47–50^ Studying the impact of *H. pylori* status on beta diversity, we observed that if *H. pylori* is present in the gastric mucosa it gains a clear predominance, which alters the gastric microbial composition in *H. pylori*–infected individuals.^47–51^ Most reports show that *H. pylori*–positive and *H. pylori*–negative individuals’ microbiota are mainly dominated by the same phyla but with different percentages of relative abundance.^34,47,52^ *H. pylori*–positive individuals have a higher abundance of Proteobacteria, probably resulting from the contribution of *H. pylori*, while there is a lower abundance of Actinobacteria, Bacteroidetes, and Firmicutes.^34,43,44,48,49,52^ Only one human study discusses the taxonomic differences between *H. pylori*–positive and *H. pylori*–negative groups after *H. pylori* sequence reads were removed.^34^ When *H. pylori* sequences were left out of the analysis, the phylotype evenness and diversity of *H. pylori*–positive individuals were higher than that of *H. pylori*–negative individuals. Further examination of the phylum distribution of all non–*H. pylori* phylotypes of individuals based on *H. pylori* status revealed no gross differences in taxonomic patterns. Martin et al. assessed the impact of *H. pylori* on the preexisting gastric microbial community in a rhesus macaque model. There was no significant difference in the average relative abundance of non-*Helicobacter* taxa in pre- and post-inoculation samples after removing *Helicobacter* reads.^53^ The rhesus model suggests the rhesus gastric microbial community is largely stable despite the immunological and physiological changes that occur due to *H. pylori* infection. In human studies, the gastric microbial diversity changes associated with *H. pylori* seem to be reversible to some degree. Eradication of *H. pylori* infection may increase the diversity of gastric microbiota.^44,54–56^

### Helicobacter pylori, the gastric microbiome, and gastric cancer

*H. pylori* is well-recognized as a class I carcinogen for gastric cancer.^57,58^ Infection initiates chronic gastric inflammation and destroys the hydrochloric acid-secreting glands of the stomach, ultimately leading to the precancerous changes of atrophic gastritis (AG) and intestinal metaplasia (IM).^5,59,60^ Although *H. pylori* infection is known to precipitate this cascade, cohort studies show that only 1%–2% of *H. pylori*–infected individuals develop gastric cancer.^61^ Moreover, the point of no return that leads to gastric cancer in the carcinogenesis cascade is reportedly associated with IM and dysplasia, independent of *H. pylori* status.^62^ *H. pylori* virulence, host genetics, and environmental factors all contribute to the development of gastric cancer.^63^

Before *H. pylori* was discovered in 1982, it had repeatedly been shown that bacteria multiply during gastric diseases, such as peptic ulcer diseases and gastric cancer. Hewetson et al. seem to have been the first to study material taken directly from the stomach during surgery.^64^ They took cultures from the stomach in 36 cases and a variety of bacteria were isolated. They concluded that 72% of the cases with gastric ulcers were positive for bacteria, compared with 17% of the cases without gastric ulcers. Later studies consistently showed the percentage of sterile stomach samples was lower in patients with gastric ulcers than in patients with duodenal ulcers, which is probably associated with the acidity and mucosal atrophy in the stomach.^65–67^ Several studies have investigated the bacteriology of patients with gastric cancer and found that patients with gastric carcinoma have higher bacterial counts and are colonized with higher numbers of different species than patients with other gastric diseases.^65–68^ Oropharyngeal or intestinal commensals (*Streptococcus, Bifidobacterium, Lactobacillus, Veilonella, Klebsiella, Escherichia, Pseudomonas, Neisseria, Staphylococcus*, and *Bacillus*) were reported to be associated with gastric cancer.^65,68^ The results of culture-based studies associated with gastric disease in English literature are summarized in Table 1.^64–70^ It has been hypothesized that the hypochlorhydria associated with AG allows for bacterial overgrowth in the stomach, and this may play a role in gastric carcinogenesis.^71^ However, research on the microbiota and gastric cancer remained relatively unexplored until the development of NGS.

**Table 1.** Studies analyzing the role of gastric microbiota in gastric diseases using culture-based methods

Studies	Participants	Sample type	Culture findings		Remarks
Hewetson et al., 1904^64^	36 gastric dilatation cases with or without ulceration	Gastric contents	Firmicutes, Proteobacteria, Actinobacteria; yeast	*Streptococci, Bacillus coli, Micrococci, Streptococcus pyrogenes, Bacillus subtilis, Staphylococcus albus (epidermiditis), Sarcinae* (family clostridiaceae), *Bacillus proteus, bacillus, Torula* (yeast)	The positive culture rate was 72% in patients with GU compared with a 17% positive culture rate in patients without GU.
Rosenow et al., 1915^69^	18 GU	Gastroduodenal ulcer or regional lymph glands	Firmicutes, Proteobacteria, Actinobacteria; yeast	*Streptococci, Streptococcus viridans, Staphylococci*, Gram-positive bacilli, Gram-negative bacilli, colon bacilli, *Bacillus welchii*, Diphtheroid bacilli, spore-forming bacilli; yeast	Of the 18 patients, 16 had positive culture results from the ulcer base. The almost constant occurrence of *Streptococci* in PUD suggests *Streptococci* (usually *viridans*) may play a role in the pathogenesis of ulcers.
Seley et al., 1941^113^	16 GC, 6 GU, 18 DU, 29 secondary ulcers	Mucosa obtained by surgery	Firmicutes, Proteobacteria, Actinobacteria; yeast	*Staphylococcus haemolyticus, S. viridans*, non-hemolytic *Streptococci, Clostridium welchii, B. coli, Enterococcus, B. friedlanderi (Klebsiella pneumoniae), Staphylococcus aureus, S. albus, B. proteus, Bacillus pyocyaneus, B. subtilis, Neisseria catarrhalis, Corynebacterium hodgkinii, Saccharomyces*	Positive cultures in 93.7% of GC, 83.3% of GU, 36.6% of DU, and 37.9% of secondary peptic ulcers; pathogenic bacteria (*S. haemolyticus, S. viridans*, non-hemolytic streptococci, *C. welchii*, and *Bact. coli*) were isolated from 88% of the GC samples vs. 30% of the GU samples.
Barber et al., 1946^65^	27 GU, 12 DU, 10 GC	Swab on stomach mucosa	Firmicutes, Proteobacteria, Actinobacteria; yeast	*S. viridans*, non-hemolytic streptococci, coliform bacilli (*Bact. coli, proteus, B. fecalis alcaligenes), S. albus, Neisseriae, Streptococcus pneumoniae*, Diphteroid bacilli, *S. aureus, S. pyogens*, Lactobacilli; *M. albicans*	Bacteria were isolated from the stomach ± duodenum in 90% of the patients with GC; GU cases had a lower proportion of positive culture results (55%), while swabs were sterile from all 12 cases of DU. *M. albicans*, non-hemolytic streptococci, and coliform bacilli were isolated from patients with normal or high gastric acidity. All other bacteria were isolated only from cases with achlorhydria.
Cregan et al., 1953^66^	10 PUD, 8 GC	Gastric juice	Firmicutes, Proteobacteria	*S. mitis*, *Streptococcusacidominimus, Streptococcus MG, Streptococcus uberis, Streptococcus salivarius, Streptococci, S. pyogenes*, β-hemolytic streptococci, not Group A, B, B or G, **Serratia* liquefaciens, S. aureus, Streptococcus lactis, Staphylococcus sarophyticus; Lactobacillus* spp., *Bacillus spp., C. welchii, Bact. coli*, Bact. Intermediate type I, Bact. aerogenes type I & II, Paracolon spp., *Pseudomonas spp., H. influenzae; Candida spp.*	The bacterial load in the stomach was higher in patients with GC, compared with patients without GC, and is probably related to gastric acidity. Oral or fecal commensal flora were usually found in the gastric juice of patients with GC.
Gatehouse et al., 1978^67^	49 DU, 14 GU, 35 GC	Gastric juice	Firmicutes, Proteobacteria, Actinobacteria, Bacteroidetes; yeast	*Lactobacilli, S. viridans, Micrococci, Streptococci fecalis*, Diphtheroids, *Escherichia coli, Neisseria* spp. *Clostridium* spp., *Bacteroides* spp., *Hemophilus* spp., *S. albus, Bifidobacteria, Proteus* spp., non-hemolytic Streptococci, *S. aureus, K. aerogenes, Anerobic streptococci, Veillonella* spp., β-hemolytic streptococci; yeasts	The gastric juice was sterile in the healthy controls, in 67% of DU, in 7% of GU, and in 0% of GC samples. Oropharyngeal commensals were frequently isolated in the gastric juice. The microflora of gastric aspirate is associated with gastric pathology and gastric pH. Patients with GC had higher bacterial counts and higher numbers of different bacterial species.
Sjöstedt et al., 1985^68^	10 healthy, 10 GC	Gastric juice	Firmicutes, Proteobacteria, Actinobacteria, Bacteroidetes; yeast	*Staphylococcus, Neisseria, Streptococcus, Bifidobacterium, Lactobacillus, Veilonella, Klebsiella, Escherichia, Pseudomonas, Bacillus, Bacteroides*	Patients with GC harbored the most microorganisms in the stomach and the highest number of species. The cancer patients had more non-oropharyngeal species.
Sjöstedt et al., 1987^114^	23 GC	Gastric juice, tumor, and non-tumor	Firmicutes, Proteobacteria, Actinobacteria, Fusobacteria, Bacteroidetes	*Micrococci, Staphylococci, Streptococci, Hemophilus Neisseria, Bifidobacteria, Lactobacillus, Enterococci*, enteric Gram-negative bacteria, *Veilonella, Fusobacteria, Leptotrichia, Bacteroides, Clostridium spp*.; yeast	The gastric pH correlated with the total number of microorganisms in the gastric juice; significantly higher numbers of different strains and anaerobic microorganisms colonized the tumor compared to the gastric mucosa.
Kato et al., 2006^70^	1 gastritis, 1 GU, 5 early GC, 1 gastric adenoma, 1 dyspepsia	Gastric juice and biopsy	Firmicutes, Proteobacteria, Bacteroidetes, Fusobacteria	*Streptococcus spp., Staphylococcus spp., Neisseria spp., Bacillus spp., Veillonella spp., Bacteroides fragilis, Fusobacterium, Lactobacillus spp., Peptostreptococcus anaerobius*	Impaired gastric acid secretion associated with long-term *H. pylori* infection enabled non-Helicobacter bacteria to colonize the human stomach. Higher bacterial load (100-fold) correlated with higher pH.

GU: gastric ulcer; GC: gastric cancer; PUD: peptic ulcer disease.

**Figure 1.**
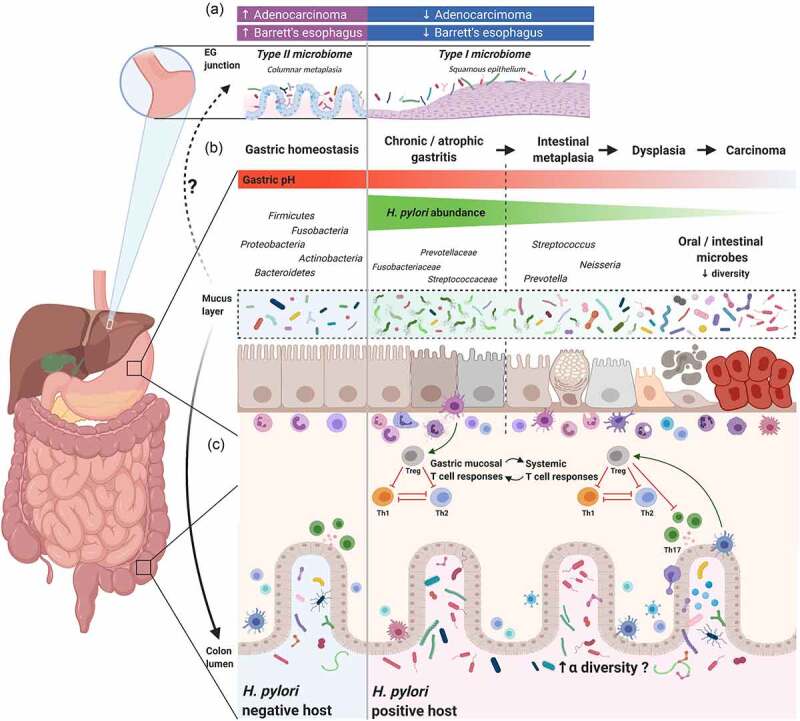
The interplay between *Helicobacter pylori* and gastrointestinal (GI) microbiota

Dicksved et al. conducted one of the first DNA-based studies investigating the gastric microbiota in patients with gastric cancer using terminal restriction fragment length polymorphisms in combination with 16S rRNA gene cloning and sequencing.^72^ They found an enrichment of *Streptococcus, Lactobacillus, Veilonella*, and *Prevotella*, and a low abundance of *H. pylori* in ten patients with gastric cancer. This was followed by 16 studies that assessed the role of the gastric microbiota in gastric cancer (Table 2, Figure 1(a)).^44,46,73–86^ Most of these studies observed a reduction in bacterial diversity or richness in the shift from non-atrophic gastritis to gastric cancer, while five studies showed different results. Dicksved et al., Wang et al., and Jo et al. did not find a significant difference in diversity indices between gastric cancer patients and controls.^72,75,76^ However, two of the studies were small in size and underpowered, which made it difficult to detect potential differences in microbiota diversity between groups.^72,75^ Eun et al. reported an increase in microbial diversity from gastritis to cancer, but provided this result without a supporting statistical analysis.^74^ Castaño-Rodríguez et al. utilized an RNA rather than DNA-based analysis and their findings cannot be directly compared with other studies.^78^ In addition to sample size and differences in methodology, Cocker et al. and Stewart et al. concluded that the discrepancies in the published studies may result from demographic characteristics, including gender, age, *H. pylori* infection status, and ethnicity.^80,87^

**Table 2.** Summary of studies examining the relationships between gastric cancer and gastric microbiota

Author, year	Sample size	Country	Microbial diversity	*H. pylori* in GC	Taxon differences
Dicksved et al., 2009^72^	10 GC, 5 dyspepsia	Sweden	No difference	N/A	N/A
Aviles-Jimenez et al., 2014^73^	5 NAG, 5 IM, 5 GC	Mexico	α-diversity: NAG > IM > GC	N/A	↑*Lactobacillus, Lachnospiraceae* from NAG, IM, to GC; ↓Saccaribacteria (TM7), Porphyromonas, *Neisseria* in GC
Eun et al., 2014^74^	11 GC, 10 IM, 10 CG	Korea	↑α-diversity in GC vs. IM & CG (not significant)	N/A	↑ *Streptococcus, Lactobacillus, Veillonella*, and *Prevotella* in GC
Wang et al., 2016^75^	6 GC, 6 CG	China	No difference in α-diversity	N/A	↑*Lactobacillus, Escherichia-Shigella, Nitrospirae, Burkholderia fungorum*, and uncultured *Lachnospiraceae* in GC
Jo et al., 2016^76^	34 GC, 29 control	Korea	No difference in α- and β-diversity	N/A	↑Actinobacteria, *Staphylococcus epidermidis* in GC; ↑ nitrosating/nitrate‐reducing bacteria in GC (not statistically significant)
Yu et al., 2017^77^	80 cardia GC, 80 non-cardia GC	80 China, 80 Mexico	↓α-diversity in GC (Chinese cohort), but not in Mexican cohort	↓	↓Proteobacteria, ↑Bacteriodetes, Firmicutes, Fusobacteria, and Spirochetes in tumor (Chinese cohort)
Li et al., 2017^44^	8 healthy control, 9 gastritis, 9 IM, 9 GC	Hong Kong	↑ Shannon index in GC vs. gastritis ↓ phylogenetic diversity in GC vs. IM	N/A	↑*Flavobacterium, Klebsiella, Serratia marcescens, Stenotrophomonas, Achromobacter, Pseudomonas, Delftia, Ralstonia, Rhizobium, Elizabethkingia meningoseptica, Methyloversatiis, Gp4, Cytophagaceae* in GC
Castaño‐Rodríguez et al., 2017^78^	12 GC, 20 dyspepsia	Singapore and Malaysia	↑α-diversity in GC	N/A	↑*Lactococcus, Veilonella*, and Fusobacteriaceae (*Fusobacterium* and *Leptotrichia*) in GC
Hsieh et al., 2018^79^	9 gastritis, 7 IM, 11 GC	Taiwan	N/A	↓	↑*Burkholderia, Enterobacter*, and *Leclercia, Clostridium, Fusobacterium* in non-GC; ↑*Lactobacillus* in GC, *C. colicanis* and *F. nucleatum* represent diagnostic markers for GC
Ferreira et al., 2018^46^	discovery cohort: 81 gastritis, 54 GC	Portugal	↓α-diversity in GC	↓	↑*Citrobacter, Clostridium, Lactobacillus, Achromobacter*, and *Rhodococcus* in GC patients
Coker et al., 2018^80^	21 superficial gastritis, 23 atrophic gastritis, 17 IM, 20 GC	China	↓α-diversity in GC and IM vs. SG	N/A	↑oral flora, *Peptostreptococcus stomatis, Streptococcus anginosus, Parvimonas micra, Slackia exigua* and *Dialister pneumosintes* in GC; ↓Vogesella, Comamonadaceae and *Acinetobacter* in GC
Hu et al., 2018^81^	6 GC, 5 CG	China	↓bacterial richness in GC, but not Shannon diversity index	N/A	↑ *Neisseria, Alloprevotella, Aggregatibacter, Streptococcus mitis* and *Porphyromonoas endodontalis* in GC; ↓*Sphingobium yanoikuyae* in GC
Liu et al., 2019^82^	276 GC	China	↓α-diversity in GC	↓	↓*Prevotella copri* and *Bacteroides uniformis;* ↑*Prevotella melaninogenica, Streptococcus anginosus* and *Propionibacterium acnes*
Gunathilake et al., 2019^83^	288 GC, 288 control	Korea	↓α-diversity in GC	↑	↑*Prevotella copri* and *Propionibacterium acnes* in GC; ↑ *Lactococcus lactis* in controls
Park et al., 2019^84^	55 GC, 19 IM, 62 CG	Korea	N/A	N/A	↑*Rhizobiales* in IM vs. gastritis; ↑*Cyanobacteria* in *H. pylori*–negative CG patients
Wu et al., 2020^85^	18 GC, 32 superficial gastritis	China	↓α-diversity in GC	N/A	↑*Dialister, Helicobacter, Lactobacillus, Rhodococcus, Rudaea* and *Sediminibacterium* in GC; 18 genera were depleted in GC; ↓*Bradyrhizobium* and *Mesorhizobium* in tumor vs. non-tumor
Gantuya et al., 2020^86^	48 GC, 120 control (20 healthy, 20 gastritis, 40 atrophy, 40 IM)	Mongolia	α-diversity: normal > IM > GC > gastritis and atrophy	↓	↑*Enterococcus, Lactobacillus, Carnobacterium, Glutamicibacter, Paeniglutamicibacter, Fusobacterium*, and *Parvimonas* in GC

GC, gastric cancer; CG, chronic gastritis; NAG, non-atrophic gastritis; IM, intestinal metaplasia; N/A, not available.

(A) Case-control and epidemiology studies demonstrated *H. pylori* infection is inversely associated with Barrett’s esophagus and esophageal adenocarcinoma. Studies suggest that the healthy esophagus is associated with a Type I microbiome, which is dominated by *Streptococcus*, while Barrett’s esophagus is associated with a Type II microbiome, containing a lower relative abundance of *Streptococcus* and a greater proportion of Gram-negative bacteria. Whether *H. pylori* directly or indirectly influences the esophageal microbiome, and the relationship between *H. pylori*, Barrett’s esophagus, esophageal adenocarcinoma, and the esophageal microbiome still needs to be elucidated. (B) Schematic plot presentation of the influence of *H. pylori* on gastric and colonic microbiota. In healthy, non-inflamed mucosa, the gastric mucosa comprises a thick layer of mucus, which serves as a protective barrier and as a highly diverse, specialized niche for colonization of gastric microbiota. In *H. pylori*–positive patients with chronic (atrophic) gastritis, *Helicobacter* dominates the gastric mucosa, resulting in reduced microbial diversity. Other bacteria, like Streptococcaceae, Fusobacteriaceae, and Prevotellaceae, may be present to a lesser extent. After a long period of co-infection and co-colonization, combined with the presence of risk factors that determine the gastric dysbiotic parietal cell loss with an increase in pH, the innate immune response and gastric microbiota interactions promote the progression of pre-neoplastic lesions. In the later stages of carcinogenesis, ranging from intestinal metaplasia to gastric adenocarcinoma, a reduction or depletion of *H. pylori* is seen in the gastric mucosa. In gastric cancer, microbial diversity is reduced, and oral or intestinal-type bacteria are enriched. (C) In chronic *H. pylori* infections, the *H. pylori*–experienced dendritic cells retain a semi-mature phenotype and induce immunosuppressive regulatory T cell (Treg) differentiation, rather than Th1 or Th17 cells from naive Th0 cells.^93,136,137^ Tregs produced in the gastric mucosa are trafficked to other lymphoid tissues in distant organs to exert a systematic immunoregulatory effect that influences the pathogenesis of various immune-related diseases, such as asthma and inflammatory bowel disease.^138,139,140,141^ The immunoregulatory effect induced by *H. pylori* strengthens the host’s resilience against microbiome perturbations and may result in increased colonic microbiota diversity. Additionally, chronic *H. pylori* infection alters the acidic environment in the stomach, permitting more microorganisms to pass through the gastric acid barrier and colonize the distal gut. The gut microbiota may also induce Tregs and in turn, regulate *H. pylori*–associated immune responses, which includes complex crosstalk between *H. pylori* and colonic microbiota.

Previously published studies show reduced *H. pylori* abundance in tumor tissue compared with adjacent non-neoplastic areas,^77,79,82,88^ suggesting that bacteria other than *H. pylori* may play a role in the development of gastric cancer. To determine whether changes in gastric microbiota play a role in the development of gastric cancer or are secondary to the changes in the gastric environment, studies of rodent model systems have helped to identify important drivers and modiﬁers of diseases related to the microbiome. Studies using the insulin-gastrin (INS-GAS) transgenic mouse model demonstrated that mice infected with *H. pylori* together with the colonization of commensal flora developed more severe gastric lesions and had earlier development of GI intraepithelial neoplasia compared with *H. pylori*–infected germ-free INS-GAS mice, highlighting the idea that the gastric microbiota may participate in the cascade of events leading to gastric cancer following *H. pylori* infection.^89,90^ Although a consensus has not yet been reached regarding the dominant bacteria potentially involved in human gastric cancer development, an increase in several oral and intestinal commensal bacteria has been reported in several studies. Ferrairi et al. reported the enrichment of *Achromobacter, Citrobacter, Lactobacillus, Clostridium, Rhodococcus*, and *Phyllobacterium* in gastric cancer microbiota.^46^ Using a co-occurrence/co-exclusion network analysis, Coker et al. identified the enrichment of *Peptostreptococcus stomatis, Streptococcus anginosus, Parvimonas micra*, and *Slackia exigua* in gastric cancer and determined that *Dialister pneumosintes* was crucial to the gastric cancer occurrence network, and these findings were successfully validated in the Inner Mongolian cohort.^80^

The majority of the reported studies are based on cross-sectional comparisons of individuals with and without histological changes in the gastric mucosa. This approach only provides a unique snapshot in time, which does not allow us to derive information about gastric carcinogenesis. A recent systemic review failed to find significant differences in microbiota profiles between individuals with superficial gastritis, atrophic gastritis, and IM.^36^ Defining a gastric cancer microbial signature without considering the underlying mechanism of the ensuing dysbiosis provides a limited perspective with limited therapeutic potential. A recent study carried out in Shandong, China analyzed 102 paired gastric biopsy samples taken before and one year after *H. pylori* eradication.^56^ Sung et al. demonstrated *Roseburia* and *Sphingomonas* were depleted in patients with persistent inflammation one year after *H. pylori* eradication. The emergence and persistence of gastric atrophy and IM one year following *H. pylori* eradication were associated with a cluster of oral bacteria comprising *Peptostreptococcus, Streptococcus, Parvimonas, Prevotella, Rothia*, and *Granulicatella*. This study supports the hypothesis that the presence of *H. pylori* provides various microbiome niches contributing to gastric cancer development. A larger multicenter, multicultural, prospective study focusing on the gastric microbiota during gastric carcinogenesis is warranted to validate the results and to explore underlying mechanisms.

## *Helicobacter pylori* and colonic microbiota

The microbial component of the human digestive tract is at its highest in the colon, with nearly a 10^7^-fold increase in number compared with the stomach.^91^ The GI tract is a complex and dynamic network with interplay between intestinal epithelial cells, the immune system, food, host metabolism, and commensal microbes. Numerous studies have attempted to define the microbial signatures of various diseases and possible microbial therapeutic interventions. Considering the commensal microbiota and the host form a unique entity in a continuum along the GI tract, any changes in the GI microenvironment may influence the homeostasis of the entire system. The studies described in the previous section reveal that *H. pylori* colonization has a great impact on the gastric microbiome. Nevertheless, the effect of *H. pylori* on colonic microbiota remains largely unexplored.

### Helicobacter pylori and colonic microbiota in rodent models

Theoretically, *H. pylori* may influence colonic microbiota through crosstalk with the host immune system or through changes in the local gastric environment. Kienesberger et al. infected neonatal C57Bl/6 mice with *H. pylori* strain PMSS1 at four or six weeks of age. The study demonstrated that *H. pylori* not only influences the gastric microbial community structure but also has systemic effects and alters the distal gut microbiota.^92^ Studies have shown *H. pylori* infection acts as an immunoregulator of regulatory T cell induction through the downregulation of IL-18 in *H. pylori*–infected mice, which results in immunotolerance and the facilitation of *H. pylori* persistence.^92,93^ *H. pylori* may regulate microbial composition in the distal intestine in a similar fashion. The most significant route of impact would possibly be through *H. pylori*–induced hypochlorhydria in the stomach. It is plausible that hypochlorhydria may promote the entrance of acid-sensitive bacteria into the distal GI tract, resulting in the alteration of the colonic microbiome. Heimesaat et al. investigated the GI microbiota changes in Mongolian gerbils after 14 months of infection with *H. pylori* and reported distinct shifts in microbiota composition of the distal uninflamed GI tract of wildtype *H. pylori*–infected animals.^94^ Gastric immunopathology with reduced gastric acid and hypergastrinemia during *H. pylori* infection has been put forward as a hypothetical explanation for the distal gut microbiota changes. Additionally, reduced leptin and ghrelin secretion in *H. pylori*–infected individuals may indirectly influence the GI microenvironment by modulating gastric acid secretion and the immune response, which in turn alters the microbial composition of the GI tract.^92,95–97^

### Helicobacter pylori and colonic microbiota in humans

Compared to studies investigating the effect of *H. pylori* on human gastric microbiota, relatively few studies have addressed the influence of *H. pylori* on colonic microbiota (summarized in Table 3, Figure 1(b)).^47,54,98–112^ Most studies have focused on the consequences of *H. pylori* eradication therapy.^98–101^ Earlier studies using culture-based approaches^98-100^ or fluorescent in situ hybridization^99^ suggested different compositions of gut microbiota among *H. pylori*–infected and uninfected individuals. Bühling et al. and Myllyluoma et al. concluded that the total number of anaerobes was significantly lower in *H. pylori*–positive individuals compared with *H. pylori*–negative individuals.^98,99^ The advent of culture-independent approaches, high-throughput sequencing coupled with advances in computational methods, have enabled genome-wide dissection of *H. pylori* and gut microbiota interactions. Eleven studies have assessed the gut microbiota in *H. pylori*–infected individuals (Table 2). The majority of these studies were in Asian populations and children were included in three studies. Microbiota composition was assessed from fecal specimens by DNA amplification (in nine studies) or by shotgun sequencing (in one study).^54^ One study used reverse‐transcribed RNA for 16S rRNA gene sequencing to assess microbial communities in fecal and colon biopsy specimens.^47^

**Table 3.** Summary of studies examining the effect of *Helicobacter pylori* infection on colonic microbiota

Detection method	Author, year	Participants	Age	Country	α-diversity	Findings
Cultivation	Bühling et al., 2001^98^	51 *H. pylori* vs. 27 control	Adult	Germany	N/A	↓Anaerobes in *H. pylori* patients; ↓Enterobacteria, *Clostridium innocuum* and *Veillonella* spp. in *H. pylori* patients; ↑Lactobacilli, esp. *Lactobacillus acidophilus* in *H. pylori* patients
	Myllyluoma et al., 2007^99^	39 *H. pylori* vs. 19 control	Adult	Finland	N/A	↓*Clostridium histolyticum* and anaerobes in *H. pylori* patients
	Yang et al., 2012^100^	38 *H. pylori* vs. 38 matched control	Child	Taiwan	N/A	↓*Bifidobacterium, Bifidobacterium:Escherechia coli* ratio; ↑*E. coli*
	Benavides-Ward et al., 2018^103^	28 *H. pylori* vs. 28 control	Child	Peru	N/A	↑Proteobacteria, Firmicutes and *Prevotella* in *H. pylori* patients
Next-Generation Sequencing	Chen et al., 2018^101^	70 *H. pylori* vs. 35 control	Adult	China	↑ richness (Sobs index)	22 genera and 38 bacterial species differ; predicted metabolic pathways differ
	Iino et al., 2018^102^	226 *H. pylori* vs. 524 control (111 non-AG, mild AG, severe AG)	Adult	Japan	N/A	↑*Lactobacillus* in severe AG vs. mild & non-AG
	Gao et al., 2018^104^	24 *H. pylori* vs. 22 non*−H. pylori* (negative control + past infection)	Adult	China	Non-significant ↑ Shannon index in gastritis and metaplasia	No differences in β-diversity; some genera differ; ↓Bacteroidetes, ↑Firmicutes and ↑*Proteobacteria* associated with *H. pylori–*related gastric lesion progression
	Osaki et al., 2018^105^	5 *H. pylori*–infected children and 13 family members	Child and adult	Japan	N/A	No differences in β-diversity and Firmicutes/Bacteroidetes ratio; some genera differ
	Iino et al., 2019^106^	226 *H. pylori* vs. propensity score matched control	Adult	Japan	↑	β-diversity differs; some genera differ; ↑*Streptococcus* in severe AG vs. non-AG in *H. pylori* patients
	Wang et al., 2019^107^	128 *H. pylori* vs. 158 control	Adult	China	No differences	β-diversity differs; some genera differ
	Dash et al., 2019^108^	12 *H. pylori* vs. 48 control	Adult	United ArabEmirates	↑	No difference in β-diversity; some genera differ
	He et al., 2019^54^	17 *H. pylori* vs. 7 control	Adult	China	↑	β-diversity differs; ↑Proteobacteria, Actinobacteria, and Acidobacteria; some genera differ
	Vasapolli et al., 2019^47^	6 *H. pylori*, 15 non−*H. pylori*	Adult	Germany	N/A	No differences in β-diversity
	Yang et al., 2019^109^	50 *H. pylori*, 42 control	Child	China	No differences	β-diversity differs; some genera differ
	Frost et al., 2019^110^	212 *H. pylori* vs. 212 control	Adult	Germany	↑	β-diversity differs; some genera differ; more enterotype 2 in *H. pylori* patients; *Bacteroides, Barnesiella, Alistipes*, and *Fusicatenibacter* negatively associated with HpSA load
	Cornejo-Pareja et al., 2019^111^	40 *H. pylori* vs. 20 control	Adult	Spain	↓	β-diversity differs
	Zhou et al., 2020^112^	22 *H. pylori* vs. 23 control	Child	China	No differences	No differences in α-diversity and β-diversity; some genera differ

AG, atrophic gastritis; N/A, not available; HpSA, *Helicobacter pylori* stool antigen

Except for one study,^111^ most reports show higher^54,101,106,108,110^ or unchanged^47,104,105,107,109^ alpha diversity indices from the gut microbiota of *H. pylori*–infected individuals compared to *H. pylori*–negative controls. The two largest cohorts enrolled 214 *H. pylori*–infected Japanese participants and 212 *H. pylori–*infected German participants and both showed higher alpha diversity compared with matched *H. pylori–*negative controls,^106,110^ while Wang et al. reported no differences in alpha diversity indices between 128 *H. pylori*–infected individuals and 158 *H. pylori–*negative controls.^107^ High microbial diversity is usually regarded as an indicator of a healthy gut microbiome, while a reduction in diversity is associated with poorer health or diseases. The reason why *H. pylori* infection is associated with higher diversity is not fully understood. It may reflect the fact that *H. pylori* is ancestral and has co-evolved with humans over tens of thousands of years.^4^ It has been suggested that *H. pylori* infection strengthens the host’s resilience against microbiome perturbations or GI infections, which results in higher fecal microbiota diversity in hosts.^110^ Another possible explanation for this phenomenon is that chronic *H. pylori* infection alters the acidic environment in the stomach, permitting more microorganisms to pass through the gastric acid barrier and reach the distal gut.

Seventeen studies reported differences when comparing fecal microbiota compositions of *H. pylori*–infected and non-infected individuals. Among thirteen studies using NGS technology, six studies observed differences in beta diversity between *H. pylori–*infected and non-infected populations,^54,107,109–111^ while five studies showed no differences in fecal microbiota composition.^47,104,105,108,112^ It is possible that the small sample size of the studies left them statistically underpowered, and potential differences in microbiota composition between groups would be difficult to detect. Chen et al. conducted the first study employing NGS technology to assess fecal microbiota composition in patients infected with *H. pylori*.^101^ The study revealed a significant difference of 22 bacterial genera between *H. pylori*–positive and negative populations. However, the differential taxa of colonic microbiota between infected and uninfected groups have not been well characterized in the published literature (Supplementary Table 1). A higher abundance of *Haemophilus, Howardella, Gemella*, and *Streptococcus*, alongside a lower abundance of *Pseudoflavonifractor, Fecalibactrium, Ruminococcus*, and *Eubacterium ventriosum* in fecal samples has been reported in *H. pylori*–infected patients (Supplementary Table 1). The inconsistency in differential taxa in fecal microbiota associated with *H. pylori* infection may reflect the heterogeneity of age, ethnicity, dietary habits, and gastric pathology in the study populations. Iino et al. demonstrated that *Streptococcus* was significantly more abundant in feces of *H. pylori*–infected individuals with severe gastric atrophy, compared with that in *H. pylori*–infected individuals without atrophic gastritis.^106^ This suggests *H. pylori* infection and the extent of gastric mucosal atrophy may affect the composition of the gut microbiota in Japanese populations. In addition, Gao et al. showed that alterations in the fecal microbiota, especially the dominant phyla of Bacteroidetes, Firmicutes, and Proteobacteria, may be associated with *H. pylori*–related gastric lesion progression in a Chinese population.^104^ The impacts of gastric pathology severity on fecal microbiota require further investigation because the evidence is still limited.

### *Colonic microbiota and consequences of* H. pylori *eradication*

**Figure 2.**
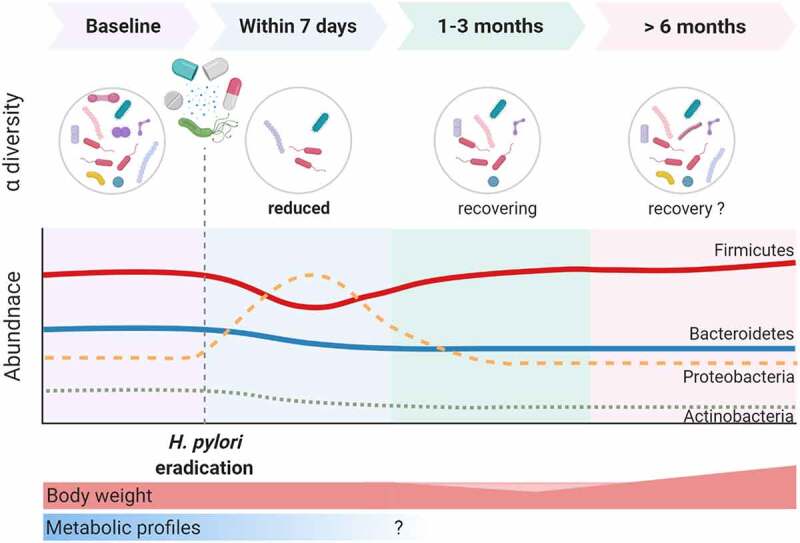
The impact of *Helicobacter pylori* eradication on the gut microbiome

Antibiotics break the homeostasis of gut microbiota and result in short-term alterations in the healthy gut microbiota and potentially long-lasting changes in its composition and function.^115^ One of the ways that *H. pylori* influences the colonic microbiome would be through *H. pylori* eradication therapies. Jakobsson et al. revealed that a short‐term antibiotic treatment for *H. pylori* eradication delivered a profound insult to the GI flora and resulted in a perturbed oral and colonic microbiome observed one week after treatment and persisting up to four years later.^116^ Several articles have reported short‐term and long‐term changes in gut microbiota after *H. pylori* eradication and are reviewed and summarized in Table 4 and Figure 2.^54,55,101,112,116–124^ Most of the studies used triple therapy or bismuth quadruple therapy. The short-term changes in gut microbiota after these therapies have been reported in nine studies using culture-independent approaches.^54,101,112,119–124^ All of these studies showed significant perturbations in the diversity and composition of gut microbiota immediately after *H. pylori* eradication. Long‐term changes (over six months) were reported in seven studies, although most had low numbers of cases. Of the seven studies that assessed the long‐term changes in gut microbiota at least six months after *H. pylori* eradication, most reported full recovery of bacterial diversity. However, He et al. reported higher alpha diversity after eradication therapy in children,^54^ and the largest cohort from Liou et al. demonstrated reduced alpha diversity one year after eradication therapies in patients that received regimens containing metronidazole (quadruple therapy or concomitant therapy).^122^ Additionally, some studies observed notable changes in abundance at the genus level over six months following *H. pylori* eradication. A recent meta-analysis compared the taxa changes at three different follow‐up periods after *H. pylori* eradication.^125^ In general, Actinobacteria populations decreased compared with baseline levels. Proteobacteria populations increased during short‐term follow‐up and then returned to baseline levels. *Enterobacteriaceae* and *Enterococcus* increased in the short‐term and interim follow‐up. However, there were no consistent changes in Firmicutes, Bacteroidetes, *Bifidobacterium*, or *Lactobacillus*, probably due to sample size, ethnicity, and eradication regimens.

Significant perturbation of the diversity and composition of gut microbiota develops soon after *H. pylori* eradication. The microbial diversity recovers during the follow-up, but there is not yet sufficient data to confirm the changes in alpha diversity that occur at the long-term follow-up. There is a reduction in Actinobacteria, relative to baseline, throughout the follow-up. Proteobacteria have a higher relative abundance at the short-term follow-up, which then returns to normal. Only during the long-term follow-up was a reduction in Bacteroidetes and a rise in Firmicutes evident.

**Table 4.** Summary of studies examining the impact of *Helicobacter pylori* eradication therapy on the gut microbiota

Authors, year	Number of cases	Regimen used for HP eradication	Sample type	Methods	Short-term changes (2–3 months)	Long-term changes (at least 6 months)
Jakobsson et al., 2010^114^	6	PPI, amoxicillin, clarithromycin for 7 days	feces	16S rRNA gene using 454-based pyrosequencing and T-RFLP	N/A	Diversity recovered; some notable changes in genera
Yap et al., 2015^115^	17	PPI, amoxicillin, clarithromycin for 7 days	feces	16S rRNA gene sequencing using Illumina MiSeq	N/A	No significant diﬀerences in α-diversity and β-diversity; ↓Bacteroidetes; some notable changes at genus levels
Oh et al., 2016^116^	23 (non-probiotics: 11; probiotics: 12)	PPI, amoxicillin, clarithromycin, ± probiotics for 7 days	feces	16S rRNA gene sequencing	N/A	N/A
Yanagi et al., 2017^117^	20	PPI, amoxicillin, clarithromycin for 7 days	feces, without DNAstabilizer	16S rRNA gene sequencing	No significant diﬀerences in α-diversity; ↓F:B ratio	N/A
Hsu et al., 2018^118^	11	PPI, bismuth, metronidazole, tetracycline for 14 days	feces, without DNA stabilizer	16S rRNA gene sequencing using Illumina MiSeq	↓α-diversity; the relative abundances of all phyla restored at week 8	No significant diﬀerences in α-diversity and β-diversity; some notable changes at genus levels
Chen et al., 2018^101^	70 (non-probiotics: 35; probiotics: 35)	PPI, bismuth, furazolidone, amoxicillin, ± probiotics for 14 days	feces	16S rRNA gene sequencing using Illumina MiSeq	α-diversity not completely recovered at week 8; ↓F:B ratio at week 8	N/A
Gotoda et al., 2018^119^	8 children	Vonoprazon, amoxicillin, clarithromycin for 7 days	feces	16S rRNA gene sequencing using Illumina MiSeq	No significant diﬀerences in α-diversity and β-diversity; two students showed significant changes	N/A
Liou et al., 2019^120^	234 (80 triple; 73; concomitant; 77 bismuth quadruple)	PPI-amoxicillin-clarithromycin for 14 days, concomitant for 14 days, bismuth quadruple for 10 days	feces, with DNA stabilizer	16S rRNA gene sequencing using Illumina MiSeq	α-diversity not completely recovered in concomitant and quadruple at week 8	α-diversity not fully recovered in concomitant and quadruple therapy; some notable changes at the genus levels
Martín-Núñez et al., 2019^121^	40	PPI, amoxicillin, clarithromycin, for 10 days	feces	16S rRNA gene sequencing using Illumina MiSeq	↓α-diversity; ↓Actinobacteria at week 8	N/A
Hsu et al., 2019^122^	12	Reverse hybrid for 14 days	feces	16S rRNA gene sequencing using Illumina MiSeq	α-diversity and β-diversity restored at week 8; relative abundance of all genera restored	No significant diﬀerences in α-diversity, β-diversity, or relative abundance of bacteria at genus level
He et al., 2019^54^	10	Bismuth quadruple for 14 days	feces	16S rRNA gene sequencing using Illumina MiSeq	No significant difference in phyla at week 4	Significantly higher α-diversity; β-diversity differs; ↑Firmicutes, Actinobacteria; ↓Proteobacteria, Bacteroidetes; notable changes in some genera
Guo et al., 2019^55^	34	Bismuth quadruple for 10 days	feces	16S rRNA gene sequencing using Illumina MiSeq	N/A	No significant diﬀerences in α-diversity; β-diversity differs; ↑Firmicutes; ↓Bacteroidetes; ↑F:B ratio; notable changes in some genera
Zhou et al., 2020^112^	22 children and 23 control	Bismuth quadruple for 10 days	feces	16S rRNA gene sequencing using Illumina MiSeq	↓α-diversity at week 6; β-diversity restored at week 6	No significant diﬀerences in α-diversity, β-diversity, and relative abundance of bacteria at genus level

PPI, proton pump inhibitors; F:B ratio, Firmicutes:Bacteroidetes ratio; HP, *Helicobacter pylori*; N/A, not available; T-RFLP, terminal-restriction fragment length polymorphism

In summary, the human digestive tract is a complex ecosystem and *H. pylori* infection alters not only gastric acidity but also host-microbe interactions, which may result in changes in colonic microbiome composition. Antibiotics are a double-edged sword. The antimicrobial agents (including bismuth) used for *H. pylori* eradication and gastric cancer prevention have direct effects on the colonic microbiota during short-term and possibly also long-term evaluations.

### *Helicobacter pylori, gut microbiota, and* H. pylori*–related extragastric disease*

*H. pylori* has been associated with multiple extragastric diseases, such as cardiovascular diseases, neurological diseases, obesity, metabolic syndromes, and chronic immune-mediated disorders.^126^ The underlying pathogenic mechanisms are not yet understood. The gut microbiota are involved in nutrient absorption, metabolism, and development and stimulation of the host immune system and digestive tract. It is hypothesized that gut microbiota may play a role in *H. pylori*–associated diseases. A large-scale cross-sectional study in Japan demonstrated significantly higher low-density lipoprotein levels and significantly lower high-density lipoprotein levels in men who were *H. pylori* seropositive, compared with *H. pylori* seronegative men.^127^ Studies have shown a significant increase in body mass index and body weight after eradication of *H. pylori*,^122,128^ which may be partially explained by the restoration of ghrelin secretion, the relief of dyspepsia,^129^ or a reduced Bacteroidetes-to-Firmicutes ratio.^55^ In contrast to weight gain, studies showed improvement in insulin resistance, fasting glucose, total cholesterol, and triglyceride levels following eradication therapy.^122,130^ The improvement in these metabolic parameters may be attributed to gut microbiota alteration. He et al. demonstrated *H. pylori* infection resulted in alterations of gut microbiota and metabolic phenotypes consistent with those observed in a high-fat diet mouse model.^131^ This study suggests there is complex crosstalk between *H. pylori* and the microbiota. Treatment of *H. pylori* may be beneficial for patients with impaired glucose tolerance in addition to diet control.

As for autoimmune disorders, there is growing evidence that *H. pylori* may protect hosts from chronic immune-mediated disorders such as asthma,^9^ atopic disease,^132^ and inflammatory bowel disease,^133,134^ which have been previously attributed to the activation of Th1 cells and inhibition of the Th2 allergic response by *H. pylori*.^10^

An animal study showed that gut microbes belonging to the families *Turicibacteraceae, Erysipelotrichaceae*, and *Desulfobirionaceae*, which have been linked to changes in the host immune response, are influenced by the presence of *H. pylori* in mice.^92^ Evidence suggested that the maturation of the human gut microbiota progresses by accruing microbes, followed by subsequent development and enrichment of the microbiome ecosystem throughout early childhood.^135^ Chen et al. identified a negative association between *H. pylori* and asthma only in the younger age group of children 3–13 years old.^9^ Malaty et al. examined the age of *H. pylori* seroconversion in a prospective cohort and suggested the peak period for newly acquired *H. pylori* infection was highest among children aged 4–5 years.^136^ Since the gut microbiome gradually develops its structure and function during childhood,^137^ further exploration is required to determine whether *H. pylori* by itself or in combination with the gut microbiota altered by infection protects the host against chronic immune-mediated illnesses. Targeted studies examining the impact of *H. pylori* during early childhood are urgently needed to help address its specific role in subsequent microbial colonization.

## Conclusions

The advances in GI microbiota research allow investigators and clinicians to explore the role of the microbiome in various diseases including, but not limited to, GI diseases. Culture-independent techniques, particularly those based on high‐throughput or NGS technology, have revolutionized our knowledge of the GI microbiota. *H. pylori*, as one of the most important microbial members of the human GI tract, has been a significant focus for a long time due to its importance within the pathophysiology of peptic ulcer disease and gastric cancer. It is undisputed that significant differences exist in the microbiota of individuals with different gastric pathology, atrophic gastritis, IM, and gastric cancer, highlighting that dysbiosis in the stomach is a dynamic process and correlates with gastric carcinogenesis. The gastric cancer microbiota has drawn researchers’ attention and has been found to be enriched with intestinal or oral taxa. However, most studies on gastric microbiota and gastric cancer development are retrospective and correlational in nature. Longitudinal and prospective studies are needed to identify the presence of specific bacterial species or microbial consortia and the underlying pathways as the microbiota changes during gastric cancer carcinogenesis. It is possible that the presence of certain changes could be used to develop biomarkers to monitor disease progression and to develop disease-modifying therapies to manipulate the gastric microbiota and prevent the risk of developing gastric cancer.

The GI tract is a complex and dynamic ecosystem with interplay between various gut mucosal cells and their defense molecules, the immune system, food particles, and resident microbes. The harsh acidic environment of the stomach serves as a gated entrance to the GI system. *H. pylori* infection reduces gastric acid and changes the gastric microenvironment, which may in turn influence subsequent GI commensal microbiota colonization. Scientific efforts have been focused on the benefits of treating and eradicating *H. pylori*, and its relative absence provides us an opportunity to investigate a more complex gut-microbial–host-immune/metabolic axis. The current investigations on the complex crosstalk between *H. pylori* and the gut microbiota are far from conclusive. Most of the studies have been association studies and the exact underlying mechanisms need to be unraveled further. Longitudinal studies with a focus on the gut microbiota and host phenotype changes during *H. pylori* infection in humans are missing, as well as studies specifically evaluating the possible long‐term effects of eradication therapies on the GI microbiota. Multiomics approaches employing shotgun sequencing or long-read sequencing technology, in combination with metabolomics, are needed to clarify the long-term implications of gut microbiota and host physiology alterations following *H. pylori* eradication. The newly acquired knowledge in this field will provide insight into host-microbial crosstalk and will make microbial-directed therapies against diseases possible.

## Supplementary Material

Supplemental Material
