# Supplementary material for: The interplay between Helicobacter pylori and gastrointestinal microbiota
Source: Gut Microbes. 2021 May 3;13(1):1909459. doi: 10.1080/19490976.2021.1909459 (PMC8096336; doi:10.1080/19490976.2021.1909459)
Supplement: Supplemental Material [file KGMI_A_1909459_SM6834.docx]

**Supplementary Table 1.** Characteristics of included articles, according to exposure ‘group’. Where studies included multiple exposures, the primary exposure of interest was used for categorisation.

| **First author, year, country** | **Study type +/- cohort name** | **Study aim/s** | ***n* (mother-infant pairs)** | **Recruitment** | **Intervention or Exposure/s** | **Outcome/s** | **Methodology, infant age at stool sample** | **Risk of bias (SIGN)** |
| --- | --- | --- | --- | --- | --- | --- | --- | --- |
| **Probiotic and prebiotic supplementation during pregnancy** | | | | | | | | |
| Abrahamsson 2009 (74)  Sweden | Prospective, randomized, double-blind, controlled trial | To assess the prevalence of *L reuteri* in stool and breast milk from mothers and infants after *L reuteri* supplementation to evaluate factors influencing levels of *L reuteri*, and to assess the influence on the microbial ecology associated with allergy | 232  Family history of atopic disease | At antenatal clinics, timing not further defined | *L reuteri* supplementation (droplets) or placebo, taken daily by pregnant women for 4 weeks before term and continued to delivery. Infants commenced the same *L reuteri* supplementation and continued daily for 1 year | 1. Prevalence of *L reuteri* in maternal and infant stool, and breast milk samples  2. Prevalence of bifidobacteria or *C difficile* in stool of infants | Culture methods  5 days  1 month  3 months  6 months  12 months | Acceptable |
| Avershina 2016 (75)  Norway  *See: Dotterud 2015* | Randomized, double-blind trial  Probiotics in Prevention of Allergy among Children of Trondheim  (ProPACT) | To assess if there is a difference in detection frequency of bacteria isolated from stool samples of infants whose mothers were supplemented with probiotic-containing milk during pregnancy compared to controls, and if supplemented bacteria are shared within mother–child pairs | 415 | Via midwives at pregnancy check-ups, <36 weeks’ gestation | Daily intake of a fermented milk supplement (*Lactobacillus rhamnosus* GG (LGG), *Bifidobacterium animalis subsp. lactis* Bb-12, and *Lactobacillus acidophilus* La-5) or heat-treated milk (placebo) from 36 weeks’ gestation until 3 months postpartum | 1. Detection frequency of LGG, L. acidophilus and Bifido-bacterium spp. in stool samples  2. Sharing of intestinal bacterial species in mother-child pairs in stool samples taken from late pregnancy to offspring at 24 months old | 16S rRNA gene sequencing  10 days  3 months  12 months | Acceptable |
| Bisanz 2015 (76)  Tanzania | Randomized controlled trial | To assess the effect of probiotic yogurt (*Lactobacillus rhamnosus* GR-1), supplemented with Moringa plant on the health and microbiota of pregnant women in Tanzania and the gut microbiota of their infants | 56 | At antenatal clinic visits | Intake of 250g of a probiotic yogurt (containing *Lactobacillus rhamnosus* GR-1 and supplemented with Moringa plant), taken 6 days a week from the time of recruitment until 1 week to 1 month postpartum | 1. Maternal anthropometry  2. Maternal vaginal and intestinal microbiome abundance and diversity  3. Maternal diet  4. Infant anthropometry  5. Infant intestinal microbiome abundance and diversity  6. Breast milk composition | 16S rRNA gene sequencing  3 days  1 week  1 month | Poor |
| Dotterud 2015 (77)  Norway | Randomized, double-blind trial  Probiotics in Prevention of Allergy among Children of Trondheim  (ProPACT) | To investigate if a probiotic supplement, given to mothers during the last 4 weeks of pregnancy and 3 months postnatally, altered the colonisation and diversity of the mothers’ and children’s intestinal microbiota | 415 | Via midwives during pregnancy check‐ups before 36 weeks’ gestation | Daily intake of a fermented probiotic milk supplement (containing *Lactobacillus rhamnosus* GG, *Bifidobacterium animalis subsp. lactis* Bb-12, and *Lactobacillus acidophilus* La-5) or heat-treated milk that contained no probiotic bacteria (placebo) daily, from 36 weeks’ gestation until 3 months postpartum | Composition and diversity of the intestinal microbiome in infants whose mothers consumed the study probiotic milk supplement compared to placebo | 16S rRNA gene sequencing, PCR^^  10 days  3 months  12 months | Acceptable |
| Enomoto 2014 (78)  Japan | Open trial | To determine if supplementation of bifidobacterial powder to mothers during pregnancy and to infants in their first 6 months of life affects the development of atopic disease and intestinal microbiome composition | 166 | Via research nurses at a hospital antenatally | Supplementation with bifidobacterial powder from 4 weeks before the expected date of delivery. After delivery, infants were given one sachet of the same bifidobacterial powder daily in breast milk, infant formula or water until 6 months | 1. Development of allergic symptoms in infants  2. Intestinal microbiome composition in mothers and infants | 16S rRNA gene sequencing  4 months  10 months | Poor |
| Gronlund 2007 (79)  Finland | Double-blind, placebo-controlled trial | To assess the association between maternal breastmilk and fecal bifidobacteria and infants’ fecal bifidobacteria, in women receiving a probiotic supplement during pregnancy compared to those who did not | 61  Family history of atopic disease | At their first visit to a maternal welfare clinic | Probiotic supplementation including *Bifidobacterium lactis* (Bb 12) and *Lactobacillus rhamnosus* (GG) from 15 weeks of gestation until a maximum of 6 months post-partum (or the end of exclusive breastfeeding) | 1. Prevalence of allergy in infants  2. Infant intestinal microbiome composition (and interaction between maternal and infant composition) | PCR^^  1 month | Acceptable |
| Gronlund 2011 (80)  Finland  *See: Gronlund 2007* | Double-blind, placebo-controlled trial | To elucidate the mother-infant association in the gut colonization of 1–6-month-old infants and to establish whether probiotics can influence this process | 80  Family history of atopic disease | At their first visit to a maternal welfare clinic | Three intervention groups:  1. *Lactobacillus rhamnosus* with *Bifidobacterium longum*  2. *Lactobacillus paracasei* with *Bifidobacterium longum*  3. Placebo | 1. Correlation of faecal bacterial counts between mothers and infants  2. Bifidobacterial diversity indexes and the mother-infant similarity indexes | PCR^^  1 month  6 months | Acceptable |
| Grześkowiak 2012 (81)  Finland, Germany | Randomized, double-blind, placebo-controlled clinical trial | To investigate the intestinal microbiota composition and structure infants who received specific probiotic combinations during early feeding either via the mother (Finland) or incorporated in early formula-feeding (Germany) | 79 (Finland)  81 (Germany)  Family history of atopic disease | At antenatal clinic visits | Finland: mothers consumed one of two probiotic products  (*Lactobacillus rhamnosus* or *Lactobacillus paracasei)* for 2 months before delivery and 2 months after delivery during breast-feeding, versus placebo  *Germany: The study intervention used (formula) was not included in this review; it did not start during pregnancy.* | Intestinal microbiota composition and structure of infants:  a) Whose mothers received probiotics during pregnancy versus placebo (Finland)  b) Who did or did not receive specific probiotic combinations directly via formula feeding (Germany) *[not included in this review]* | PCR^^, Flow cytometry - FISH°  6 months | Acceptable |
| Gueimonde 2006 (82)  Finland | Placebo-controlled trial | To characterize both the mother-infant bifidobacteria transfer at birth and the development of bifidobacteria microbiota during the first weeks of life in infants whose mothers received *Lactobacillus rhamnosus* GG or placebo during pregnancy | 53  Family history of atopic disease | At antenatal clinic visits | Mothers received *Lactobacillus rhamnosus* GG (LGG) or placebo for 2-4 weeks before delivery and until the end of the study (up to 3 weeks postpartum) | Fecal bifidobacterial composition of mothers and infants (presence of *B. adolescentis, B. breve, B. bifidum, B. catenulatum group,*  *B. infantis* and *B. longum*) | PCR^^  5 days  3 weeks | Poor |
| Ismail 2012 (83)  Australia | Randomized, double-blind, placebo-controlled trial | To investigate the effect of pre-natal administration of *Lactobacillus rhamnosus* GG on infant gut microbial diversity | 98  Family history of allergy | At antenatal clinics and via community advertising | Probiotic supplementation containing *Lactobacillus rhamnosus* GG or placebo once daily from week 36 of gestation until delivery | Faecal microbial diversity in infants | 16S rRNA gene sequencing  7 days | Acceptable |
| Korpela 2018 (84)  Finland | Randomized, double-blind, placebo-controlled trial | To determine whether probiotic supplementation could ameliorate the effects of antibiotic use or caesarean birth on infant microbiota | 428  Family history of allergy | In antenatal clinics | Mixed probiotic supplement (containing *Bifidobacterium breve*, *Propionibacterium freundenreichii* subsp. *shermanii* JS, *Lactobacillus rhamnosus* and *Lactobacillus rhamnosus* GG) or placebo, during pregnancy and in infants for 6 months | 1. Infant intestinal microbiota composition  2. Whole metagenome and metaproteome composition (sub-analysis) | 16S rRNA gene sequencing,  Metagenomic sequencing  3 months | High |
| Kukkonen 2007 (85)  Finland | Randomized, double-blind, placebo-controlled trial | To determine whether dietary supplementation with 4 probiotic strains and prebiotic galactooligo-saccharides protects infants against allergic disease and changes their intestinal microbiome | 925  Family history of allergy  131 infants provided stool samples | At antenatal clinics and via community advertising | Mixed probiotic supplement (containing *Lactobacillus rhamnosus* GG, *L rhamnosus*,  *Bifidobacterium breve* and *Propionibacterium freuden-reichii* ssp.*shermanii* JS) or placebo during pregnancy and in infants for 6 months | 1. Incidence of any allergic disease (food allergy, asthma, eczema and allergic rhinitis) and IgE-associated (atopic) disease at age 2 years  2. Eczema and IgE sensitization  3. Infant intestinal bacteria counts | Culture methods  Meconium  3 months  6 months | High |
| Niers 2009 (86)  The Netherlands | Randomized, double-blind, controlled trial  The Paediatric Anaesthesia Neurodevelopment Assessment (PANDA) study | To investigate the effect on the development of eczema during the first 2 years of life, and initial effects on early microbial colonization and immune responses | 156  Family history of allergy | Advertising campaign, ≥2 months prior to delivery | Probiotic supplementation containing *Bifidobacterium bifidum, Bifidobacterium lactis* and *Lactococcus lactis* or placebo, given to pregnant mothers and to infants during their first year of life | 1. Incidence of eczema in the first 3 months of life  2. Serum total IgE and specific IgE  3. Whole blood cultures and cytokine analysis  4. Infant intestinal microbiome composition | PCR^^  1 week  2 weeks  1 month  3 months  12 months | Acceptable |
| Parnarnen 2018 (87)  Finland | Subset of a double-blind, randomized, placebo-controlled trial | To determine whether mothers’ gut and breast milk microbiota influence the infant gut resistome and MGEs during the first 6 months of life by quantifying sharing of genes and bacteria between mothers and infants | 16  Family history of allergy | In antenatal clinics | Multi-nutrient and probiotic supplementation (containing vitamins and minerals plus *Lactobacillus rhamnosus* LPR and *Bifidobacterium longum* (or) Lactobacillus paracasei and *Bifidobacterium longum*) versus placebo (same dietary supplement without probiotics) | 1. Abundance of antibiotic resistance genes (ARGs) in breast milk and infant stool  2. Abundance of mobile genetic elements (MGEs) in breast milk and infant stool  3. Infant intestinal microbiome composition and diversity | Metagenomic analysis  1 month  6 months | Acceptable |
| Rinne 2005 (88)  Finland | Randomized, double-blind, placebo-controlled trial | To assess the impact of probiotics and breastfeeding on gut microbiota composition as characterized by bifidobacteria and lactobacilli/enterococci and humoral immune responses as indirectly assessed by circulating immunoglobulin (Ig) secreting cells | 96  Family history of allergy | In antenatal clinics | Probiotic supplementation containing *Lactobacillus rhamnosus* GG daily for 4 weeks prior to delivery, or placebo (microcrystalline cellulose) | 1. Concentration of soluble CD14 in colostrum  2. Abundance of bifidobacteria and lactobacilli/enterococci in infant stool  3.Total numbers of Ig secreting cells | FISH°  3 months  6 months  12 months | Acceptable |
| Rinne 2006 (89)  Finland | Randomized, double-blind, placebo-controlled trial | To determine whether probiotics administered prenatally and for 6 months postnatally affect gastro-intestinal symptoms, crying and the compositional development of the gut microbiota through infancy | 132  Family history of allergy | In antenatal clinics | Probiotic supplementation containing *Lactobacillus rhamnosus* GG daily for 4 weeks prior to delivery, or placebo (microcrystalline cellulose) and for 6 months postnatally (mothers or infants) | 1. Numbers of gastrointestinal symptoms (vomits, stools)  2. Duration of crying/fussing  3. Infant stool bacterial numbers for *Bifidobacterium, Lactobacillus/Enterococcus,* *Bacteroides* and *Clostridium* | FISH°  6 months  12 months | Acceptable |
| Rutten 2015 (90)  The Netherlands  *See: Niers 2009* | Randomized, double-blind, controlled trial  The Paediatric Anaesthesia Neurodevelopment Assessment (PANDA) study | To assess the long-term effects of probiotics on the composition and diversity of gut microbiota over time in infants at risk for atopic disease and to investigate the differences in microbiota between children who did and did not develop atopic disease | 123  Family history of allergy  86 infants provided stool samples | Advertising campaign, at least 2 months prior to delivery | Probiotic supplementation containing *Bifidobacterium bifidum, Bifidobacterium*  *lactis* and *Lactococcus lactis* or placebo, given to pregnant mothers and to infants during their first year of life | Intestinal microbiota composition and diversity | 16S rRNA gene sequencing,  Flow cytometry,  FISH°  1 week, 2 weeks  1 month, 3 months  12 months | Acceptable |
| Shadid 2007 (91)  Germany | Randomized, double-blind, placebo-controlled pilot | To test how supplementation with galactooligosaccharides (GOS) and long chain fructooligo-saccharides (lcFOS) in pregnancy affects maternal and neonatal gut microbiota and foetal immunity | 33  32 infants provided stool samples | In antenatal clinics prior to third trimester | Prebiotic mixture (containing 45.45% short-chain GOS and 4.91% lcFOS) or placebo (maltodextrin) | 1. Bifidobacteria and lactobacilli counts in the maternal and neonatal gut  2. Gut microbiome diversity  3. Cord blood analyses for fetal lymphocyte subsets and cytokine secretion | FISH°  5 days  20 days  6 months | Acceptable |
| **Antibiotic use during pregnancy and/or labour** | | | | | | | |  |
| Aloisio 2016 (97)  Italy | Case-control | To evaluate the main effects of IAP^ on the gut microbiome composition of newborns | 20 | After birth, in a NICU` | Maternal GBS* positivity, treated with IAP^ during delivery versus no GBS or IAP^ | Stool microbial composition, richness and diversity | 16S rRNA gene sequencing  7 days | Acceptable |
| Arboleya 2015 (98)  Spain | Case-control | To assess the establishment of the intestinal microbiota of very low birthweight preterm infants compared to healthy full-term infants and evaluate the impact of delivery mode and antibiotic use | 40 | After birth, in a NICU` | 1. Delivery mode  2. IAP^ exposure | Infant stool microbial composition | 16S rRNA gene sequencing, PCR^^  1-2 days, 10 days  1 month  3 months | Acceptable |
| Arboleya 2016 (99)  Spain  *See: Arboleya et al 2015* | Case-control | To assess the establishment of the intestinal microbiota of very low birthweight preterm infants compared to healthy full-term infants and evaluate the impact of delivery mode and antibiotic use on the composition of and genes expressed within the infant intestinal microbiota | 40 | After birth, in a NICU` | 1. Delivery mode  2. IAP^ exposure | 1. Infant stool microbial composition  2. Infant intestinal production of short chain fatty acids  3. Infant faecal antibiotic resistance genes | 16S rRNA gene sequencing, gas chromatography, functional inference analysis  2 days, 10 days  1 month  3 months | Acceptable |
| Azad 2016 (100)  Canada | Prospective birth cohort  Canadian Healthy Infant Longitudinal Development (CHILD) study | To determine the impact of IAP^ on infant gut microbiota, and to explore whether breastfeeding modifies these effects | 198 | At antenatal clinics in a mother’s second trimester | Maternal IAP^ exposure (vaginal, elective caesarean or emergency cesarean delivery) versus no IAP^ exposure (vaginal delivery) | Stool microbial community structure, taxonomic composition, richness and diversity | 16S rRNA gene sequencing  3 months  12 months | Acceptable |
| Corvaglia 2016 (101)  Italy | Case-control | To evaluate the effect of IAP^ and feeding mode on gut microbiota in healthy term infants | 84 | After birth | 1. Maternal IAP^ for GBS  2. Mode of feeding (exclusive breastfeeding versus mixed/formula feeding) | Infant stool abundance of selected microbial groups (*Lactobacillus* spp, *Bifidobacterium* spp and *Bacteroides fragilis* group) | PCR^^  7 days  1 month | High |
| Fallani 2010 (92)  Europe (UK, Italy, Spain, Sweden, Germany) | Prospective cohort  INFABIO project | To determine the faecal microbiota composition of infants with different lifestyle characteristics and correlate this to country of origin, mode of delivery, feeding method and perinatal antibiotic treatment | 606 | After birth | 1. Country of birth  2. Perinatal antibiotics  3. Delivery mode  4. Infant feeding method (exclusive breastfeeding, formula feeding, or mixed) | Infant stool microbial composition | FISH°  6 weeks | Acceptable |
| Imoto 2018 (102)  Japan | Cross-sectional pilot | To investigate factors related to bifidobacterial colonization in early infancy, focusing on maternal antimicrobial use at delivery | 33 | At routine 1-month infant check-ups | Maternal IAP^ exposure at delivery versus no IAP^ exposure | Stool microbial composition, bifidobacterial abundance and diversity | 16S rRNA gene sequencing  1 month | Acceptable |
| Jauréguy 2004 (103)  France | Case-control | To compare patterns of acquisition of gastrointestinal bacterial flora in infants born to mothers treated with IAP due to GBS, compared to infants born to untreated mothers | 50 | Unclear | Maternal GBS* positivity, treated with IAP^ during delivery versus no GBS or IAP exposure | 1. Stool microbial composition  2. Antibiotic susceptibility | Culture methods  3 days | Acceptable |
| Jia 2018 (104) [abstract only]  China | Case-control | To obtain a longitudinal view of the gut microbial establishment in preterm versus full term vaginally delivered infants, and to identify key clinical factors that affect this | 101 | Unclear | 1. IAP^ exposure  2. Gestational age | Stool microbial composition and diversity | 16S rRNA gene sequencing  1 day – 3 months  Multiple samples | Abstract – insufficient detail available |
| Lee 2014 (93)  Korea | Prospective cohort  The Cohort for Childhood of Asthma and Allergic Diseases (COCOA) | To evaluate whether caesarean delivery, prenatal exposure to antibiotics and susceptible genotypes act additively to promote atopic disease by affecting the development of the gut microbiota in infancy | 412  11 infants provided stool samples | At antenatal appointments (36 weeks’ gestation) | 1. Family history of atopic disease (susceptive genotype) versus no family history  2. Birth mode (vaginal versus caesarean delivery)  3. Prenatal exposure to antibiotics versus no exposure | 1. Development of atopic disease in infancy  2. Diversity of intestinal microbiota | 16S rRNA gene sequencing  6 months | Acceptable |
| Mazzola 2016 (105)  Italy  *See: Aloisio 2016* | Cohort | To examine the impact of maternal IAP^ and mode of feeding on infant faecal microbiota composition | 26 | After birth, in a NICU` | 1. Infant stool microbial composition  2. Mode of feeding (exclusive breastfeeding versus mixed feeding) | Stool microbial composition and diversity | 16S rRNA gene sequencing, PCR  7 days  1 month | Acceptable |
| Nogacka 2017 (106)  Spain | Case-control | To evaluate the impact of IAP^ on the establishment of the gut microbiota in the vaginally delivered, term, healthy newborn | 40 | After birth | 1. Maternal GBS* positivity, treated with IAP^ during delivery versus no GBS or IAP exposure  2. Mode of feeding (exclusive breastfeeding versus formula feeding) | 1. Infant intestinal microbiota composition and diversity  2. Infant intestinal production of short chain fatty acids  3. Infant faecal antibiotic resistance genes | 16S rRNA gene sequencing, gas chromatography, PCR^^  2 days, 10 days  1 month  3 months | Acceptable |
| Pozo-Rubio 2013 (94)  Spain | Prospective cohort  The PROFICEL study | To assess the associations between early environmental factors, lymphocyte subsets, and intestinal microbiota composition in infants at familial risk for coeliac disease (CD) | 55  Family history of CD  44 infants provided stool samples | Before delivery, unclear regarding further timing | 1. Delivery mode  2. Gender  3. Prenatal antibiotic exposure (during pregnancy/IAP^)  4. Mode of feeding  5. Infections  6. Infant antibiotic therapy  7. Rotavirus vaccination | 1. Lymphocyte subsets  2. Infant intestinal microbiota composition | PCR^^  4 months | Acceptable |
| Stearns 2017 (107)  Canada | Prospective cohort  Baby & Mi cohort | To determine the effects of IAP^ on the development of the infant gut microbiome among a low-risk population | 74 | At antenatal midwifery practices | 1. Maternal GBS* positivity, treated with IAP^ during delivery versus no GBS or IAP exposure  2. Delivery mode (vaginally or via caesarean section) | Infant intestinal microbiota composition and diversity | 16S rRNA gene sequencing  3 days, 10 days  6 weeks  4 months | Acceptable |
| Zhang 2019 (95)  USA | Prospective cohort  The Nurture Study | To examine associations of prenatal antibiotics with infant weight and adiposity measures at 12 months and the infant intestinal microbiome at 3 and 12 months | 454  68 infants provided stool samples | Via antenatal clinics at 20-36 weeks’ gestation | Prenatal antibiotic exposure | 1. Infant weight and adiposity at 12 months  2. Infant intestinal microbiota composition | 16S rRNA gene sequencing  3 months  12 months | Acceptable |
| Zou 2018 (96)  China | Case-control | To investigate the effects of prenatal antibiotic exposure and its intensity on the development of the gut microbiota in preterm infants | 28 | After birth | 1. Prenatal antibiotic exposure and intensity of treatment (≤7 days or >7 days)  2. Delivery mode (vaginally or via caesarean section)  3. Gestational age  4. Feeding mode | Infant intestinal microbiota composition and diversity | 16S rRNA gene sequencing  7 days  14 days | Acceptable |
| **Mother’s pre-pregnancy weight status / gestational weight gain** | | | | | | | |  |
| Baumann-Dudenhoeffer 2018 (109)  USA | Prospective birth cohort | To study pre- and postnatal determinants of infant microbiome development, including feeding mode, formula ingredients, maternal antibiotic exposure, and maternal GWG ¨ | 30 mothers and twins (60 infants) | Unclear | 1. Maternal pre-pregnancy anthropometry and GWG  2. Maternal exposure to antibiotics during pregnancy/labour  3. Infant feeding practices, food group and nutrient intake, drinking water sources  4. Prebiotic intake | 1. Infant intestinal microbiome composition and diversity  2. Functional pathway enrichment | Whole meta-genome shotgun sequencing  Multiple time points from 0-8 months | Acceptable |
| Campoy 2018 [abstract only] (113)  Spain | Observational cohort | To test whether gut microbial composition and functionality at 6 months of age is associated with maternal pre-pregnancy BMI^#^ | 68 | Not defined in abstract | Maternal pre-pregnancy BMI^#^ (not defined in abstract) | Intestinal microbial composition and function | 16S rRNA gene sequencing, meta-proteomics, glycosyl hydrolase activities  6 months | Abstract – insufficient detail available |
| Cerdó 2018 [abstract only] (114)  Spain | Prospective observational cohort  Subset of the PREOBE cohort study | To investigate the impact of maternal pre-pregnancy BMI^#^ on infants’ gut microbiome and neurodevelopment | 122  46 infants provided samples at 6 months | At antenatal clinic visits during weeks 12-34 of pregnancy | Maternal pre-pregnancy BMI^#^ – normal weight (18.5 ≤ BMI ≤ 25 kg/m2) or obese (BMI ≥30 kg/m2) | 1. Intestinal microbial composition and function  2. Infant neurodevelopment | 16S rRNA gene sequencing  6 months | Acceptable |
| Collado 2010 (122)  Finland | Prospective birth cohort  Subset of Pre-, Peri- and Postnatal Programming and Origins of Disease: Early Targeting the Epidemics of Allergy and Over-weight (NAMI) | To assess the relationship between maternal pre-pregnancy BMI^#^ and gestational weight gain on the infant microbiota | 42 | At antenatal clinic visits if <17 weeks pregnant | 1. Maternal pre-pregnancy BMI^#^ (overweight or obese, defined as ≥25kg/m2, compared to women with a BMI ≤ 25 kg/m2)  2. Maternal gestational weight gain, defined as excessive if exceeding the Institute of Medicine (IOM) pregnancy weight gain recommendations | Intestinal microbiome composition in infants born to mothers who (1) had a “healthy” pre-pregnancy BMI compared to those classified as overweight or obese, and (2) exceeded gestational weight gain recommendations according to pre-pregnancy BMI, compared to those whose mothers did not gain excessive weight | FISH°, PCR^^  1 month  6 months | Acceptable |
| Lemas 2016 (115)  USA | Prospective observational cohort | 1. To identify early differences in intestinal microbiota in infants born to obese compared with normal-weight mothers  2. To explore relations between human milk hormones (leptin and insulin) and the taxonomic and functional potentials of the infant intestinal microbiome | 30 | At antenatal clinic visits | Maternal pre-pregnancy BMI^#^ category (normal weight BMI ≤ 25 kg/m2 or obese ≥30kg/m2) | 1. Infant intestinal microbiota composition, diversity and metagenomics profile, including short chain fatty acid concentrations  2. Human milk leptin and insulin concentrations  3. Infant body composition  4. Infant feeding practices | 16S rRNA gene sequencing, Whole meta-genome shotgun sequencing  2 weeks | Acceptable |
| Mueller 2016 (116)  Brazil | Prospective observational cohort | To examine the role of maternal pre-pregnancy BMI^#^ in the assembly of the intestinal microbiome of neonates delivered vaginally versus by elective Cesarean section without membrane rupture | 74 | At antenatal clinic visits | Maternal pre-pregnancy BMI^#^ status (normal weight (< 25 kg/m2) or overweight or obese (≥ 25kg/m2) | Infant intestinal microbiota community structure and functional differences in metabolic signalling and energy regulation | PCR^^, 16S rRNA gene sequencing, in silico meta-genome prediction  1-2 days (first stool after meconium) | Acceptable |
| Robinson 2017 (119)  USA | Prospective observational cohort | To examine associations of maternal GWG¨ with infant fecal microbiota composition, richness and diversity | 84 | Through a primary care group practice (during pregnancy) | Maternal GWG¨, divided into three groups: ≤11.9 kg, 12.0–14.9 kg, and ≥15.0 kg | Infant faecal microbiota profile / composition, bacterial community richness and diversity | 16S rRNA gene sequencing  ~ 4 months | Acceptable |
| Singh 2019 (120)  USA | Prospective observational birth cohort  New Hampshire Birth Cohort | To examine associations of maternal pre-pregnancy BMI^#^ and GWG¨ with the infant gut microbiome by delivery-mode strata | 355 | At antenatal clinics | 1. Maternal pre-pregnancy BMI^#^ status (normal (BMI<25, referent), overweight (BMI 25.1-30) or obese (BMI >30))  2. Maternal GWG¨ (adequate, inadequate or excess weight gain based on the Institute of Medicine recommendations) | Infant faecal microbiota composition, richness and diversity | 16S rRNA gene sequencing  6 weeks | Acceptable |
| Stanislawski 2017 (121)  Norway | Prospective observational birth cohort  The Norwegian Microbiota Study (NoMIC) | To understand whether maternal pre-pregnancy overweight/obese (OW/OB) or excessive GWG¨ are associated with differences in maternal gut microbiota at delivery or in the gut microbiota of infants | 169 | At antenatal clinics | 1. Maternal pre-pregnancy BMI^#^ (groups combined into (a) non-OW/OB: underweight/ normal weight, and (b) OW/OB: overweight/obese)  2. Maternal GWG¨ (“low”, “adequate” or “excessive” according to the Institute of Medicine’s recommendations) | 1. Maternal faecal microbiota composition and diversity  2. Infant faecal microbiota composition and diversity | 16S rRNA gene sequencing  4 days  10 days  1 month  4 months  12 months | Acceptable |
| Sugino 2019 (117)  USA | Prospective observational birth cohort  ARCH_GUT_ and BABY_GUT_ cohorts | To identify associations between maternal pre-pregnancy obesity and pregnancy or early infancy microbiotas | 39 | At their first prenatal visit | Maternal pre-pregnancy BMI^#^ (normal: 18.5-<25 kg/m2, or overweight/obese: ≥25 kg/m2) | 1. Maternal faecal microbiota composition and diversity  2. Infant faecal microbiota composition and diversity | 16S rRNA gene sequencing  ~8 days | Acceptable |
| Tun 2018 (118)  Canada | Prospective observational birth cohort  Canadian Healthy Infant Longitudinal Development (CHILD) study | To investigate the association of birth mode with microbiota in the infant gut, and whether this mediates the association between maternal and child overweight | 935 | At antenatal clinics in a mother’s second or third trimester | Maternal pre-pregnancy BMI^#^ (normal: 18.5-<25 kg/m2, overweight: 25-30 kg/m2, or obese: ≥30 kg/m2) | 1. Child BMI^#^ z scores at ages 1 and 3 years (risk of obesity)  2. Infant faecal microbiota composition and diversity | 16S rRNA gene sequencing  3-4 months | Acceptable |
| **Maternal diet and nutrient supplementation during pregnancy** | | | | | | | |  |
| Chu 2016 (127)  USA | Prospective observational cohort | To determine the effect of a maternal high-fat diet in gestation and lactation on the early infant microbiome | 157 | Antenatal appointments in early third trimester or during intra-partum | Maternal dietary fat intake (participants categorised as either consuming a “normal/ control” diet or a “high fat” diet if intake was ±1 standard deviation from the cohort mean) | Intestinal microbiome composition in infants | 16S rRNA gene sequencing  At delivery (meconium)  4-6 weeks | Acceptable |
| Lundgren 2018 (125)  USA | Prospective observational birth cohort  New Hampshire Birth Cohort Study | To examine the association of maternal diet during pregnancy with the infant gut microbiome, stratified by delivery mode | 145 | At antenatal clinics | 1. Maternal alternative  Mediterranean diet score  2. Maternal food group intake  3. Maternal fatty acid intake | Intestinal microbiome composition and clustering in infants, depending on maternal diet +/- delivery mode | 16S rRNA gene sequencing  6 weeks | Acceptable |
| Savage 2018 (126)  USA  *See: Sordillo 2017* | Randomized, controlled trial  Vitamin D Antenatal Asthma Reduction Trial (VDAART) | To determine the association between diet during pregnancy and infancy, including breast-feeding versus formula feeding, solid food introduction, and the infant intestinal microbiome | 323 | At antenatal clinics in early pregnancy | 1. Maternal diet (food or food group intake)  2. Infant feeding mode (breastfeeding, formula feeding, or solid food intake) | Infant intestinal microbiome composition, richness and diversity | 16S rRNA gene sequencing  3-6 months | Acceptable |
| Sordillo 2017 (130)  USA | Double-blind, placebo controlled, randomized trial  Vitamin D Antenatal Asthma Reduction Trial (VDAART) | To determine how prenatal and early life factors impact the gut microbiome in an ethnically diverse study population of infants | 333 | During first trimester of pregnancy, via antenatal clinics | 1. Parental allergy/asthma history versus no allergy/asthma history  2. Treatment with vitamin D supplementation and micro-nutrients (either 4000 IU Vitamin D + prenatal vitamins or 400 IU Vitamin D + prenatal vitamins) | 1. Infant intestinal microbiome composition and diversity  2. Infant 25(OH)Vitamin D concentrations in cord blood | 16S rRNA gene sequencing  3-6 months | Acceptable |
| Talsness 2017 (129)  The Netherlands | Prospective observational birth cohort  KOALA Birth Cohort Study | To determine whether maternal vitamin D supplementation, maternal plasma 25-hydroxyvitamin D (25(OH)D) concentration, or direct supplementation of the infant influences key bacterial taxa within microbiota of one month old infants | 913 | Via Steiner schools, organic food shops, magazines and health practitioners during pregnancy | 1. Maternal vitamin D supplement use  2. Maternal plasma 25(OH)D concentration  3. Infant vitamin D supplementation | Infant intestinal abundance of several key bacterial taxa (*Bifidobacterium* spp.,  *Escherichia coli, Clostridium difficile, Bacteroides fragilis* group, *Lactobacillus* spp. and total bacteria) | PCR^^  4 weeks | Acceptable |
| Urwin 2014 (128)  UK | Randomised, single-blind, controlled trial  The Salmon in Pregnancy Study (SiPS) | To investigate whether increased salmon consumption during pregnancy, maternal weight gain during pregnancy or mode of infant feeding alter the markers of gut immune defence and inflammation | 38 | Routine antenatal clinics at 12 weeks’ gestation | 1. Regular diet (rare consumption of oily fish) [controls]  2. Consumption of 2x 150g portions of salmon per week from 20 weeks of pregnancy to delivery [salmon group] | 1. Maternal intestinal microbiota composition  2. Maternal faecal secretory IgA and calprotectin concentrations  3. Infant intestinal microbiota composition  4. Infant faecal secretory IgA and calprotectin concentrations | FISH°  1 week  2 weeks  4 weeks  3 months | Acceptable |
| **Maternal diabetes** | | | | | | | |  |
| De Leon Solis 2018 [abstract only] (131)  Mexico | Unclear | To compare the fecal microbiota of children between 3 months and 3 years born to mothers with and without GDM^◊^ | 29 | Unclear | Maternal GDM^◊^ versus no diabetes | Infant intestinal microbiome composition and diversity | 16S rRNA gene sequencing  Between 3 months and 3 years | Abstract – insufficient detail available |
| Hu 2013 (66)  USA | Prospective observational cohort | To assess the diversity of the meconium microbiome and determine if the bacterial community is affected by maternal diabetes status | 23 | At regular visits to a prenatal clinic prior to their second trimester | Maternal diabetes (established diabetes, GDM^◊^ or subclinical diabetes), compared to no diabetes | Infant intestinal microbiome composition | 16S rRNA gene sequencing  2-48 hours after birth (meconium) | Acceptable |
| Su 2018 (132)  China | Prospective observational cohort | To investigate the potential effect of GDM^◊^ on newborns’ gut microbiota | 34  Infants born by caesarean section only | At antenatal clinics | 1. Maternal GDM^◊^ versus no diabetes  2. Maternal fasting glucose levels | Infant intestinal microbiome composition and diversity | 16S rRNA gene sequencing  Within 24 hours (meconium) | High |
| Wang 2018 (63)  China | Case-control | To investigate possible dysbiosis of maternal and neonatal microbiota associated with GDM^◊^ and to estimate the potential risks of the microbial shift to neonates | 486 | At prenatal visits | 1. Maternal GDM^◊^ versus no diabetes  2. Maternal fasting glucose levels | 1. Maternal saliva, stool and vaginal microbiome compositions (1–2 days before delivery)  2. Amniotic fluid microbiome composition  3. Newborn saliva microbiome composition  4. Newborn pharyngeal microbiome composition  5. Newborn meconium microbiome composition | 16S rRNA gene sequencing, whole metagenome shotgun sequencing  Within 24 hours (meconium) | Acceptable |
| **Maternal mood disorders and stress** | | | | | | | |  |
| Lee 2019 [abstract only] (136)  Korea | Prospective birth cohort  Cohort for Childhood Origin of Asthma and Allergic Diseases (COCOA) | To determine whether prenatal maternal anxiety and dietary patterns promote atopic dermatitis in offspring through the alteration of gut microbiota | Unclear | At antenatal clinics in late pregnancy | 1. Prenatal maternal anxiety  2. Prenatal maternal diet | 1. Atopic disease development at 1 year and 3 years of age  2. Infant gut microbiota composition  3. Infant short chain fatty acid production | 16S rRNA gene sequencing  6 months | Abstract – insufficient detail available |
| Togher 2017 [abstract only]  (133)  Ireland | Prospective multi-site birth cohort  Improved Pregnancy Outcomes by Early Detection  (IMPROvED) study | To assess whether maternal depressive symptomology during pregnancy is associated with adverse obstetric and infant outcomes, including a suboptimal microbiome at birth | 46 | At antenatal clinics | Maternal depressive symptoms in their second and/or third trimesters | 1. Maternal intestinal microbiome composition, richness and diversity  2. Maternal vaginal microbiome composition, richness and diversity  3. Infant intestinal microbiome composition, richness and diversity | 16S rRNA gene sequencing  1 week  2 weeks  3 weeks  3 months  5 months | Abstract – insufficient detail available |
| Vu 2018 [abstract only]  (134)  Canada | Prospective observational birth cohort  Canadian Healthy Infant Longitudinal Development (CHILD) study | To investigate the impact of maternal depressive symptoms (DS) and serotonin reuptake inhibitor (SRI) antidepressant treatment during pregnancy on microbial taxon profiles of 4-month infant stool | 1681 | At antenatal clinics in a mother’s second trimester | In mothers:  1. No DS or SRI use  2. SRI treatment with low DS levels  3. DS only  4. Both DS and SRI use  5. Pet ownership | Infant intestinal microbiome composition | 16S rRNA gene sequencing  3-4 months | Acceptable |
| Zijlmans 2015  (135)  The Netherlands | Prospective observational birth cohort  The Bibo Study | To prospectively investigate the development of the intestinal microbiota as a potential pathway linking maternal prenatal stress and infant health | 56  All infants vaginally delivered | At antenatal clinics in a mother’s third trimester | 1. Maternal prenatal stress, self-reported (“high” versus “low” stress groups)  2. Maternal prenatal salivary cortisol | 1. Infant intestinal microbiome composition  2. Infant gastrointestinal and allergic symptoms | 16S rRNA gene sequencing  7 days, 12 days  25 days  3 months, 4 months | Acceptable |
| **Maternal asthma** | | | | | | | | |
| Koleva 2017 (137)  Canada | Prospective birth cohort  Canadian Healthy Infant Longitudinal Development (CHILD) study | To determine whether lactobacilli and other microbes are reduced in the gut of infants born to an asthmatic mother | 1021 | At antenatal clinics in a mother’s second or third trimester | 1. Maternal asthma during pregnancy  2. Maternal atopic disease during pregnancy (reactions to food or environmental allergens)  3. Maternal pre-pregnancy anthropometry | Infant intestinal microbiome composition and diversity | 16S rRNA gene sequencing  3-4 months | Acceptable |
| Stokholm 2018 (138)  Denmark | Prospective observational birth cohort  Copenhagen Prospective Studies on Asthma in Childhood (COPSAC_2010_) | To analyze the nature of gut colonization patterns during the first year of life, and the associations of these patterns with later risk of asthma | 690 | Via general practitioners seeing pregnant women at antenatal visits before 26 weeks’ gestation | Maternal asthma history versus no asthma history | 1. Childhood asthma at age 5  2. Infant intestinal microbiome composition and diversity | 16S rRNA gene sequencing  1 week  1 month  12 months | Acceptable |
| **Other – maternal demographics, exposures, diseases and pregnancy complications** | | | | | | | |  |
| Bender 2016  (139)  Haiti | Case-control | To determine whether HIV infection perturbs the mother’s microbiome and breast milk human milk oligosaccharide (HMO) composition, thereby affecting the microbiome in HIV-exposed, uninfected infants | 50 | At antenatal clinic visits | Maternal HIV infection, treated by antiretroviral therapy | 1. Microbiome composition and diversity in maternal areola skin, breast milk and vagina  2. Microbiome composition and diversity in infant mouth, skin and stool | 16S rRNA gene sequencing  ~2.5 months | Acceptable |
| Cassidy-Bushrow 2016 (140)  USA | Prospective observational birth cohort  The Wayne Health, Environment, Allergy, and Asthma Longitudinal Study (WHEALS) | To determine if maternal prenatal GBS* carrier status, accounting for IAP^, was associated with differences in the early-life gut microbiota of offspring | 262  112 infant stool samples available at 1 month; 150 at 6 months | During antenatal care appointments in second trimester or later | Maternal GBS* positivity | Infant stool microbial composition and diversity | 16S rRNA gene sequencing  1 month  6 months | Acceptable |
| Chernikova 2016 (141)  USA | Observational cohort | To test the hypothesis that maternal complications significantly affect gut colonization patterns in very low birth weight infants | 9  Premature infants | Within 2 days of birth, in the NICU` | Maternal complications, including prolonged preterm premature rupture of membranes and chorioamnionitis | Infant intestinal microbiome composition and diversity | 16S rRNA gene sequencing  Meconium  Weekly thereafter, up to 14 weeks | Acceptable |
| Chu 2017 (110)  USA | Prospective observational cohort | To assess the taxonomic composition and potential function of the early neonatal microbiota across multiple body sites and up to 6 weeks of age, and determine the impact of Caesarean delivery and its potential confounders on neonatal and infant microbiota structure and function | 81 | Antenatally, by the start of the third trimester | 1. Prenatal factors (maternal age, diet, antibiotic exposure and IAP^, pre-pregnancy BMI^#^, GWG¨)  2. Mode of delivery | Maternal and infant taxonomic composition and potential metabolic function of microbiota across multiple body sites (including stool) | 16S rRNA gene sequencing, whole metagenome shotgun sequencing  Meconium  4-6 weeks | Acceptable |
| Drell 2014 (111)  Estonia | Observational cohort, part of an ongoing study on the treatment of suspected neonatal sepsis | To describe the profile of gut microbiota in extremely low birth weight (<1200 g) critically ill infants during the first two months of life | 50 | Within 3 days of birth, in the NICU` | Maternal complications, including prolonged preterm premature rupture of membranes, chorioamnionitis and antibiotic use | Infant intestinal microbiome composition and diversity | 16S rRNA gene sequencing  1 week  1 month  2 months | Acceptable |
| Gosalbes 2013 (112)  Spain | Prospective observational birth cohort  Project INMA (Infancia y Medio Ambiente, Childhood and the Environment) | To characterize the meconium microbiota in term infants, to assess whether it contributes to the future microbiota of the infants’ gastrointestinal tract, and to evaluate how it relates to lifestyle variables and atopy-related conditions | 20 | At an antenatal visit, before 13 weeks’ gestation | Maternal age, parity, social class, education level, zone of residence, smoking, antibiotic use, and intake of dairy products and organic products during pregnancy | 1. Maternal intestinal microbiome composition, richness and diversity  2. Infant intestinal microbiome composition, richness and diversity | 16S rRNA gene sequencing  Meconium  1 week  1 month  3 months  7 months | Acceptable |
| Hesla 2014 (142)  Sweden | Prospective birth cohort  Assessment of Lifestyle and Allergic Disease During Infancy (ALADDIN) cohort | To investigate how an anthroposophic lifestyle affects the infant gut microbiota | 128 | At antenatal visits to anthroposophic and conventional Maternal Child Health Centres | Maternal anthroposophic lifestyle compared to a ‘conventional’ lifestyle | 1. Maternal intestinal microbiome composition and diversity  2. Infant intestinal microbiome composition and diversity | 16S rRNA gene sequencing  6 days  3 weeks  2 months  6 months | Acceptable |
| Ho 2018 (143)  USA | Prospective observational cohort | To assess the clinical determinants of the relative abundance of feces-associated  Gammaproteobacteria in very low birth weight (VLBW) infants | 45 | After birth, in a NICU` | Maternal prenatal factors (Hispanic ethnicity, race, age, chorioamnionitis, duration of ruptured membranes, diabetes, hypertension, BMI^#^, steroid and magnesium sulfate treatment) | Infant intestinal microbiome composition | 16S rRNA gene sequencing  ~10 days  ~ 3 weeks  ~ 1 month | Acceptable |
| Hu 2017 [abstract only] (144)  USA | Prospective case-control  The MECONIUM (“Exploring MEC-hanisms Of disease traNsmission In Utero through the Microbiome") Study | To assess the impact of maternal inflammatory bowel disease (IBD) on a mother’s microbiome during pregnancy and infant microbiome development | 80 | During pregnancy, not otherwise defined | Maternal IBD | 1. Maternal intestinal, oral and placental microbiome composition and diversity  2. Infant intestinal microbiome composition and diversity | 16S rRNA gene sequencing  Multiple time points from birth (meconium) to 90 days old | Abstract – insufficient detail available |
| Levin 2016 (108)  USA | Prospective birth cohort  The Microbes, Asthma, Allergy, and Pets (MAAP) sub-study of the Wayne Health, Environment, Allergy, and Asthma Longitudinal Study (WHEALS) | To profile the bacterial gut microbiota present in neonatal and infant stool in a racially and socioeconomically diverse population-based birth cohort and to explore associations between a broad survey of pre- and post-natal environmental and sociocultural factors and early life gut microbiome composition | 298 | At antenatal clinics in a mother’s second or third trimester | Maternal prenatal factors (BMI^#^, smoking, number of previous pregnancies, African American race-ethnicity, education, income, urban residence) | Infant intestinal microbiome composition and diversity | 16S rRNA gene sequencing  1 month  6 months | Acceptable |
| Lewis 2017 (145)  Armenia, Georgia | Cohort | To investigate country of origin and birth method (differential sources of microbial exposure) and mother’s secretor-status (an influence on environmental conditions) as possible factors influencing the infant gut microbiome in two cohorts (from Armenia and Georgia) | 81 | After birth, in the maternity ward | 1. Maternal and infant country of residence  2. Maternal secretor genotype | Infant intestinal microbiome composition | 16S rRNA gene sequencing  1 week  1 month  3 months | Acceptable |
| Madan 2012 (149)  USA | Cohort | To investigate the developing intestinal bacterial microbiome in very low birth weight (VLBW) infants at risk for sepsis over time, to correlate colonization patterns and changes in diversity with clinical factors | 6 | Within 2 days of birth, in the NICU` | 1. Maternal prenatal variables, including infection requiring antibiotic treatment, premature preterm prolonged rupture of membranes, and hypertension  2. Feeding type (breast milk, fortified breast milk, or formula)  3. Infant antibiotic exposure and clinical complications | Infant intestinal microbiome composition and diversity | 16S rRNA gene sequencing  Meconium  Weekly thereafter, for a total of 8-12 weeks | Acceptable |
| Sbihi 2019 [abstract only] (146)  Canada | Prospective birth cohort  Canadian Healthy Infant Longitudinal Development (CHILD) study | To investigate the influence of a mother’s built environment during pregnancy on asthma incidence and infant stool composition and diversity | 1000 | At antenatal clinics in a mother’s second or third trimester | Mothers’ built environment during pregnancy and in the infant’s first year of life, including exposure to traffic-related air pollution (TrAP) | 1. Asthma diagnosis by 3 years of age  2. Gut maturity index from infant stool samples  3. Infant intestinal microbiome composition and diversity | 16S rRNA gene sequencing  3 months  12 months | Acceptable |
| Stearns 2017 (147)  Canada | Prospective birth cohorts  Canadian Healthy Infant Longitudinal Development (CHILD) study +  South Asian Birth Cohort (START-Canada) | To investigate the associations of ethnicity and early life exposures with the gut microbiome among 1-year-old infants | 355  173 (CHILD)  182 (START) | At antenatal midwifery practices | 1. Maternal ethnicity (groups of people who have certain racial, cultural, religious, or other traits in common)  2. Feeding type (breast milk, or formula feeding) | Infant intestinal microbiota composition and diversity | 16S rRNA gene sequencing  12 months | Acceptable |
| Tun 2017 (148)  Canada | Prospective birth cohort  Canadian Healthy Infant Longitudinal Development (CHILD) study | To determine the existence of gut microbial associations with prenatal and/or postnatal pet exposures under different scenarios, independent of siblingship and other covariates | 804 | At antenatal clinics in a mother’s second or third trimester | 1. Pet ownership (only during pregnancy or both pre- and postnatally, compared to no pet exposure at either time)  2. IAP exposure  3. Maternal race/ethnicity  4. Maternal asthma and allergy status during pregnancy  5. Type and size of household | Infant intestinal microbiome composition and diversity | 16S rRNA gene sequencing  3-4 months | Acceptable |

^ IAP: Intrapartum antibiotic prophylaxis | * GBS: group B *Streptococcus |* ^#^ BMI: Body Mass Index | ` NICU: Neonatal Intensive Care Unit | ^ ^PCR: Polymerase Chain Reaction | ° FISH: Fluorescent in situ hybridization | ¨ GWG: Gestational Weight Gain | ^◊^ GDM: Gestational Diabetes Mellitus
